# A multi-omics approach for identifying important pathways and genes in human cancer

**DOI:** 10.1186/s12859-018-2476-8

**Published:** 2018-12-12

**Authors:** H. Robert Frost, Christopher I. Amos

**Affiliations:** Department of Biomedical Data Science, Geisel School of Medicine, Dartmouth College, Hanover, 03755 NH USA

**Keywords:** Gene set testing, Pathway analysis, Cancer genomics, Driver mutations

## Abstract

**Background:**

Cancer develops when pathways controlling cell survival, cell fate or genome maintenance are disrupted by the somatic alteration of key driver genes. Understanding how pathway disruption is driven by somatic alterations is thus essential for an accurate characterization of cancer biology and identification of therapeutic targets. Unfortunately, current cancer pathway analysis methods fail to fully model the relationship between somatic alterations and pathway activity.

**Results:**

To address these limitations, we developed a multi-omics method for identifying biologically important pathways and genes in human cancer. Our approach combines single-sample pathway analysis with multi-stage, lasso-penalized regression to find pathways whose gene expression can be explained largely in terms of gene-level somatic alterations in the tumor. Importantly, this method can analyze case-only data sets, does not require information regarding pathway topology and supports personalized pathway analysis using just somatic alteration data for a limited number of cancer-associated genes. The practical effectiveness of this technique is illustrated through an analysis of data from The Cancer Genome Atlas using gene sets from the Molecular Signatures Database.

**Conclusions:**

Novel insights into the pathophysiology of human cancer can be obtained from statistical models that predict expression-based pathway activity in terms of non-silent somatic mutations and copy number variation. These models enable the identification of biologically important pathways and genes and support personalized pathway analysis in cases where gene expression data is unavailable.

**Electronic supplementary material:**

The online version of this article (10.1186/s12859-018-2476-8) contains supplementary material, which is available to authorized users.

## Background

High-throughput genomic assays have revolutionized our understanding of cancer. Projects such as The Cancer Genome Atlas (TCGA) [1] and the Catalog of Somatic Mutations in Cancer (COSMIC) [2] have collected detailed measurements of DNA sequence, mRNA expression and methylation for thousands of individual tumors across multiple cancer types. Leveraging this data, researchers have identified hundreds of genes whose somatic alterations drive human cancer [3]. Although the discovery of cancer associated mutations and genes has enabled significant advances in cancer care through the identification of new therapeutic targets and support for personalized treatment, a strictly gene or mutation-level analysis fails to capture many important aspects of cancer biology. While a small number of cancer-associated genes are commonly mutated, i.e., present in more than 10%, of tumors, and can therefore be more easily studied, there exists a much larger set of rarely mutated cancer genes spread across the human genome [4]. Adding to this complexity, the activity of cancer-associated genes can be impacted in a variety of ways including copy number variation, somatic mutations and methylation changes [5]. Capturing all of these mechanisms requires the measurement and joint analysis of multiple types of omics data. To address the complexity of the cancer genomics landscape, researchers have turned to pathway analysis methods that combine the somatic alterations or expression of multiple, functionally related, genes [6]. Cancer is fundamentally a disease of disrupted pathways in which a population of cells develops a selective growth advantage due to the altered function of pathways controlling cell survival, cell fate or genome maintenance [7]. To fully elucidate the mechanisms driving cancer, it is thus critical that researchers understand how the somatic alterations present in each tumor collectively impact these pathways.

To interpret cancer genomic data at the level of biological pathways, researchers have developed a large number of pathway analysis methods, many specifically customized for cancer data [6]. For these methods, a pathway, or gene set, refers to a group of genes whose products share a common biological function. A number of large and well maintained repositories of such gene sets now exist with some, e.g., the Gene Ontology (GO) [8], holding simple unordered genes sets and others, e.g., Reactome [9], defining pathways in terms of a complex topology of molecular interactions. In this paper, the terms pathway and gene set are used synonymously and it is assumed that topological information is unavailable. Given a collection of such gene sets, pathway analysis methods aim to identify statistically significant associations between the activity of pathway members and a phenotype of interest, e.g., cancer type or case/control status [10]. Although most pathway methods focus on population-level associations, a number of recent approaches provide pathway enrichment results at the single-subject level [11–14].

Although the pathways most commonly impacted by somatic alterations in cancer have been identified [7] and significant progress has been made developing cancer-specific pathway analysis methods [6, 11, 14–21], existing approaches have several important limitations. Many current methods focus on either gene expression data [11] or mutation data [21] and therefore fail to capture the important association between the two in cancer. The utility of methods that use only expression data is also impacted by the lack of gene expression data for many tumor samples. For methods that just use mutation data, performance is hindered by the limited number of genomic regions sequenced in many clinical settings, the overall sparsity of somatic alterations and the fact that pathways are often defined in the context of protein activity/abundance, which may not be closely linked to the mutational status of the underlying gene. Most methods that jointly analyze multiple types of omics data ignore pathway information and instead aim to identify specific mutations or mutated genes that are associated with alterations in gene expression [22, 23] or perform unsupervised clustering [24, 25]. Among the few existing pathway methods that do combine mutations with expression [14–16, 19, 20], none of them support both population-level and single subject analyzes, few provide information on both cancer-associated pathways and genes, all of them rank pathways or genes according to measures of statistical association rather than predictive performance, and most require knowledge of pathway topology, which is unavailable for many gene sets of interest, e.g., those from GO [8]. A further limitation of most existing methods is the dependence on data from matched controls, which is very limited for certain data sets [1]. Collectively, these limitations make it difficult to catalog the full set of pathways impacted in cancer along with the genes whose somatic alteration drives pathway dsyregulation. Especially challenging is the identification of the small number of pathways with potential therapeutic value from among the larger set of pathways with altered activity in human cancer.

To address these limitations, we have developed a new multi-omics pathway analysis method for cancer genomic data that aims to:

1. Identify pathways that play an important role in the pathophysiology of human cancers.
2. Identify genes whose somatic alterations are significantly associated with pathway activity.
3. Support personalized pathway analysis using only somatic alteration data for known cancer genes.

To achieve these aims, our method jointly analyzes gene expression and somatic alteration data from human tumors to build statistical models that predict the subject-level pathway activity in terms of the somatic alterations of known cancer genes. Importantly, this method does not require data from matched controls and can analyze pathways lacking topological information. This design is motivated by our hypothesis that biologically important pathways are those for which expression-level activity of the genes in the pathway relative to other cancers of the same type can be well predicted by the somatic alterations present in the tumor. To realize our method, we leveraged three important advances in cancer genomics and biostatistics, namely the development of large cancer genomics data sets that combine gene expression and somatic alteration data, e.g., TCGA [1], the creation of effective single-sample pathway analysis methods [11–14], and the development of computationally efficient estimation algorithms [26] for penalized regression models such as the LASSO [27]. The novelty of our approach lies in the combination of these three advances to build regression models that explain the variation of pathway activity within a single cancer type using gene-level measures of somatic alteration.

## Methods

**Fig. 1:**
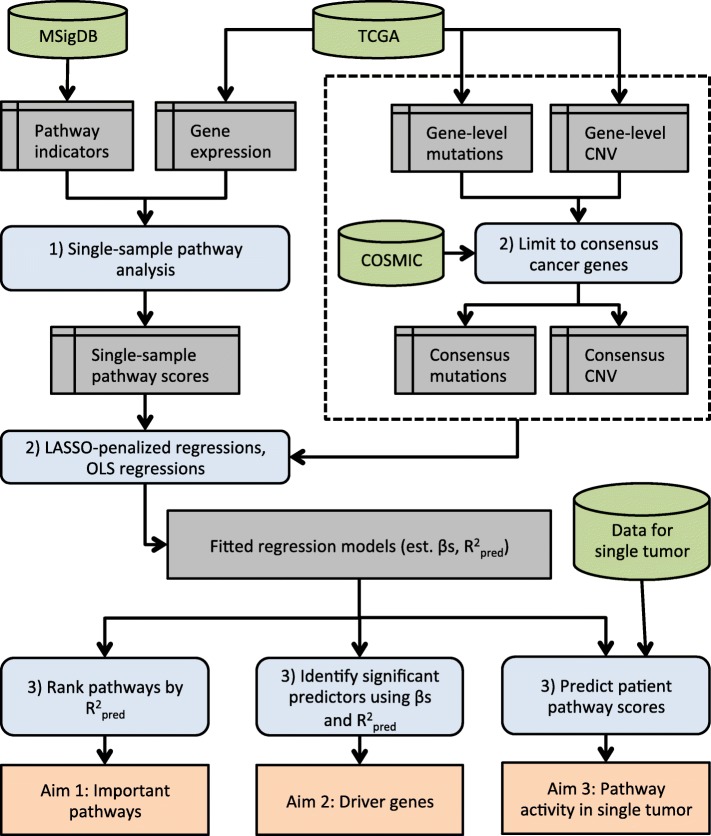
Illustration of the proposed pathway analysis method. Green cylinders represent data sources, grey boxes are intermediate data structures, blue boxes are processing steps, and orange boxes represent the analysis results associated with each of the primary aims. The numbers in each blue box correspond to one of the processing steps detailed in the “Methods” section

Our approach, illustrated in Fig. 1, finds pathways whose expression-level activity within a single cancer type is well predicted by somatic alterations. For a detailed description of the method, including data source details and relevant mathematics, please see the Additional file 1. The computational approach and code implementing this approach along with a description of its logic is provided at http://www.dartmouth.edu/hrfrost/MutPath/. This website also contains detailed information on the regression models fit using this approach. Reflecting the data sources used to evaluate our method, Fig. 1 shows TCGA as the source of cancer genomic data, the Molecular Signatures Database (MSigDB) [28] as the source of pathway definitions and the COSMIC cancer gene census [29] as the source of known cancer genes, however, the proposed method can be used with any appropriate source of tumor genomic data, any desired collection of gene sets and any relevant list of cancer-associated genes. Our approach is comprised by the following high-level steps (the step numbers match the numbered blue boxes in Fig. 1):

### Step 1. Estimate single-sample pathway activity

Our method first determines the activity of each candidate pathway within each tumor relative to other tumors of the same cancer type. This step is performed using the single-sample pathway analysis method gene set variation analysis (GSVA) [12] which takes as inputs a set of pathway definitions (i.e., a mapping of genes to pathways) and a matrix of gene expression data measured on multiple tumors of the same cancer type. We specifically utilize the variant of GSVA that identifies gene sets whose members are primarily up-regulated or primarily down-regulated. Using this data, the GSVA method generates a matrix holding pathway scores for each tumor that capture the extent to which the expression of pathway genes in that tumor deviates from the mean pathway gene expression measured in all tumor samples for that cancer type. Our motivation for performing single sample pathway analysis on just the data from a single cancer type is based on findings that variation in the expression of genes among just cancer cases is a better predictor of cancer driver genes than differential expression between cancer cases and non-cancer controls [30]. Focusing on just one cancer type also enables case-only analyses, which is important for data sets, such as TCGA, that contain little data from matched controls. It should be noted an alternate single sample gene set testing method (or variation of GSVA) can be used with our approach if such a method better captures the features of pathway activity of interest for a specific analysis (see Section 1.3. of the Additional file 1 for more details; comparative results from GSVA and ssGSEA [13] for pancreatic cancer are included in Additional file 1: Tables S24 and S25).

### Step 2. Estimate the association between pathway activity and somatic alterations

Our approach next determines how well the expression-based activity of each pathway can be predicted from gene-level somatic alterations. This step is performed via the regression of the single-sample pathway scores computed in Step 1 on gene-level indicators of non-silent somatic mutations and copy number variation (CNV) values. These models are estimated using LASSO-penalized multiple linear regression [31] with the penalty threshold and predictive performance computed via cross-validation. In particular, the predictive performance is represented by the proportion of null deviance explained by the model on the test data, which is equivalent to the predicted coefficient of determination $\left (R^{2}_{pred}\right)$ in this case. LASSO penalization is used both to identify a parsimonious set of uncorrelated predictors and to support the analysis of data sets where the number of tumor samples for a given cancer type is less than the number of predictor variables. To obtain non-shrunken coefficient estimates and the approximate statistical significance of each predictor, the penalized regression is followed by an unpenalized multiple linear regression using only those predictors with non-zero coefficients in the LASSO fit. For each pathway and cancer type combination, we fit two different regression models using this procedure. The first model uses as predictor variables non-silent somatic mutation indicators and CNV values for all genes captured in the TCGA data for the target cancer type. The second model uses somatic alteration values for the subset of TCGA genes that also belong to the COSMIC cancer gene census [2]. By comparing the models fit using only consensus cancer genes with the models fit using all available genes, it is possible to assess whether the models are capturing cancer-specific phenomena and potentially identify novel cancer-associated pathways and genes. Please see Sections 1.2 and 1.3 in the Additional file 1 for a detailed mathematical description of these regression models and the estimation procedure.

### Step 3. Interpret regression models to identify important pathways and genes

Using the regression models estimated in Step 2, one or more of the primary aims can be addressed:

*Aim 1.* To identify biologically important pathways, the pathways are ranked according to the mean $R^{2}_{pred}$ from cross-validation of the LASSO-penalized models fit using just consensus cancer gene predictors. The pathways whose activity can be well predicted by somatic alterations in cancer associated genes are deemed to be biologically important, and have potential therapeutic value, for the analyzed cancer type.

*Aim 2.* To identify genes whose somatic alteration is associated with pathway activity for a specific cancer type, the estimated coefficients for the mutation and CNV predictors in the unpenalized pathway regression models are inspected. If somatic alteration of a gene is retained as a significant predictor in the model for a specific pathway, the gene is deemed to be a potential driver for that pathway in the analyzed cancer type. A list of inferred driver genes for each cancer type can be generated by summarizing predictor significance across all pathway models while taking into account model predictive performance.

*Aim 3.* The regression models estimated in the second step enable personalized pathway analysis using just somatic alteration data for a limited number of cancer-associated genes. Specifically, given tumor-specific mutational status and CNV values for the genes with non-zero coefficients in the LASSO-penalized models, it is possible to predict the activity of each evaluated pathway in that patient. When predictions are based on the unpenalized regression models, an approximate prediction interval can also be computed.

## Results

**Fig. 2:**
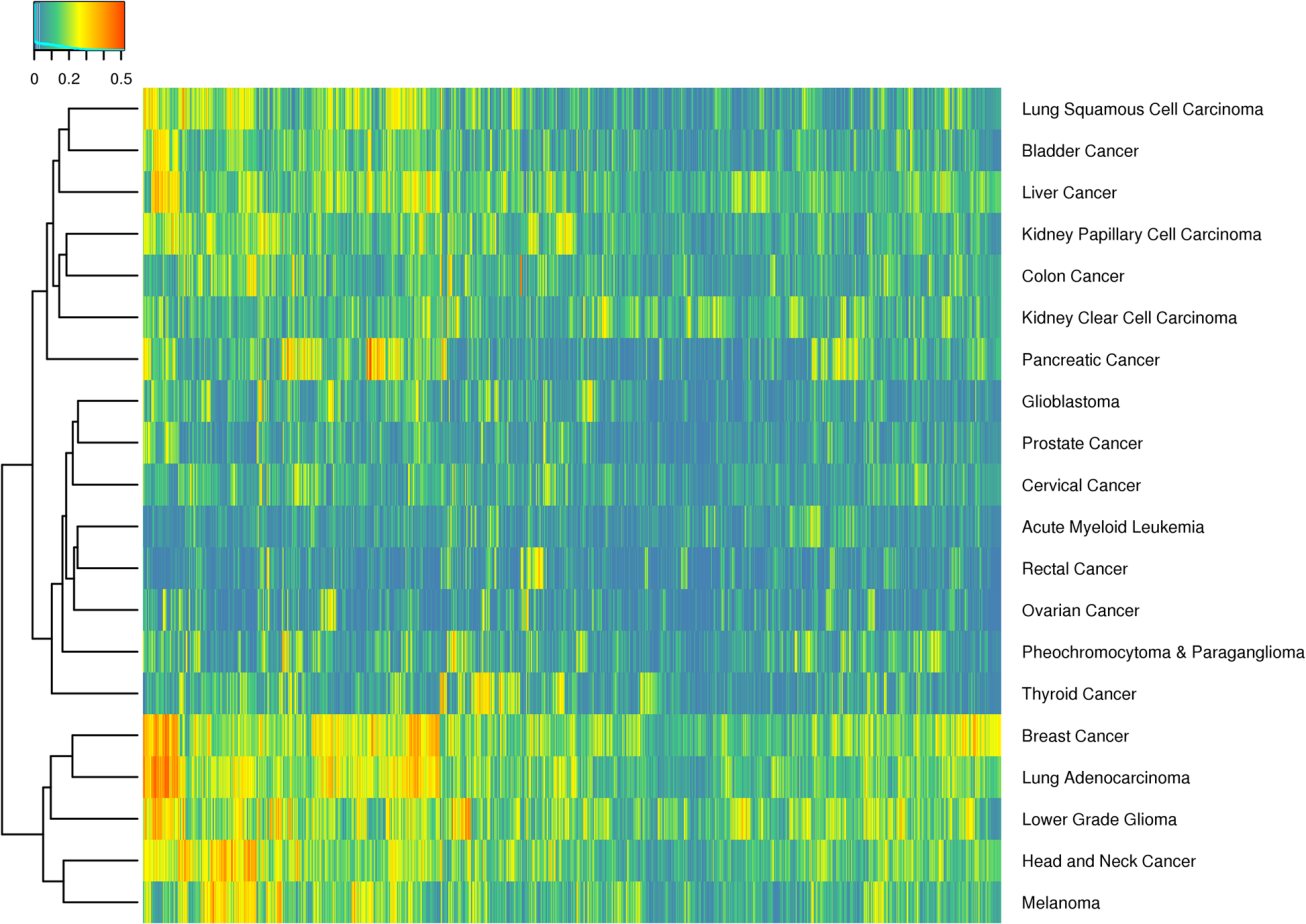
Visualization of model predictive performance. Illustration of results from the analysis of TCGA pan cancer data using the MSigDB curated canonical pathways (C2.CP) collection. Rows correspond to the TCGA cohorts and columns to MSigDB gene sets. Each cell represents a pathway and cancer type-specific model colored according to the $R^{2}_{pred}$ value. Both the columns and rows are ordered according to the output from hierarchical clustering

To evaluate our proposed method, we analyzed 20 TCGA cancer types using gene sets from the MSigDB curated canonical (C2.CP) and oncogenic signatures (C6) collections (see the Additional file 1 for details on the TCGA data sets and MSigDB collections). Figure 2 illustrates the predicted *R*^2^ values generated for the pathways in the C2.CP collection for each supported TCGA cancer type (Additional file 1: Figure S1 contains a similar heatmap for the C6 collection). Through this analysis, we aimed to answer six questions that address the overall and aim-specific effectiveness of our method:

1. Do the estimated regression models capture non-random associations?
2. Do the models capture cancer-specific phenomena?
3. (Aim 1) Does model predictive performance identify biologically important pathways?
4. (Aim 2) Can the models be used to identify cancer driver genes?
5. (Aim 3) Is model predictive performance sufficient for personalized pathway analysis?
6. Can the models be used to characterize cancer subtypes?

The following sections discuss each of these questions, and the relevant analysis results, in more detail.

### Do the estimated regression models capture non-random associations?

**Fig. 3:**
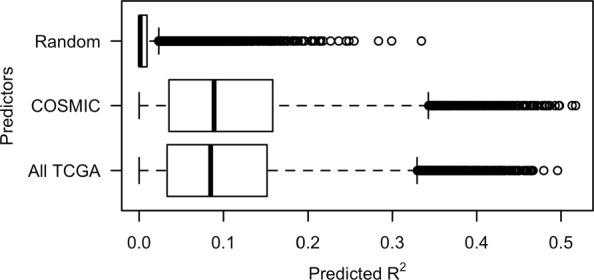
Distribution of $R^{2}_{pred}$ values. Box plot illustrating the distribution of $R^{2}_{pred}$ values from the analysis of TCGA pan cancer data using the MSigDB curated canonical pathways (C2.CP) collection. Values are shown for three different analyses: *COSMIC*: estimation of pathway regression models using somatic alteration data for COSMIC consensus cancer genes, *All TCGA*: estimation of models using somatic alteration data for all genes included in the TCGA datasets, and *Random*: estimation of models using randomized somatic alteration data for COSMIC consensus cancer genes

To answer this question, we compared the $R^{2}_{pred}$ values obtained on randomized TCGA somatic alteration data with the $R^{2}_{pred}$ values estimated using non-randomized data. Because randomized data should have no predictive power, we expected the $R^{2}_{pred}$ values for random data to be very close to zero. In contrast, we expected the $R^{2}_{pred}$ values for TCGA data to have a mean value significantly larger than zero. These expectations were confirmed by examining the distribution of $R^{2}_{pred}$ values computed using the C2.CP and C6 collections (see Fig. 3 and Additional file 1: Figure S2).

### Do the models capture cancer-specific phenomena?

**Fig. 4:**
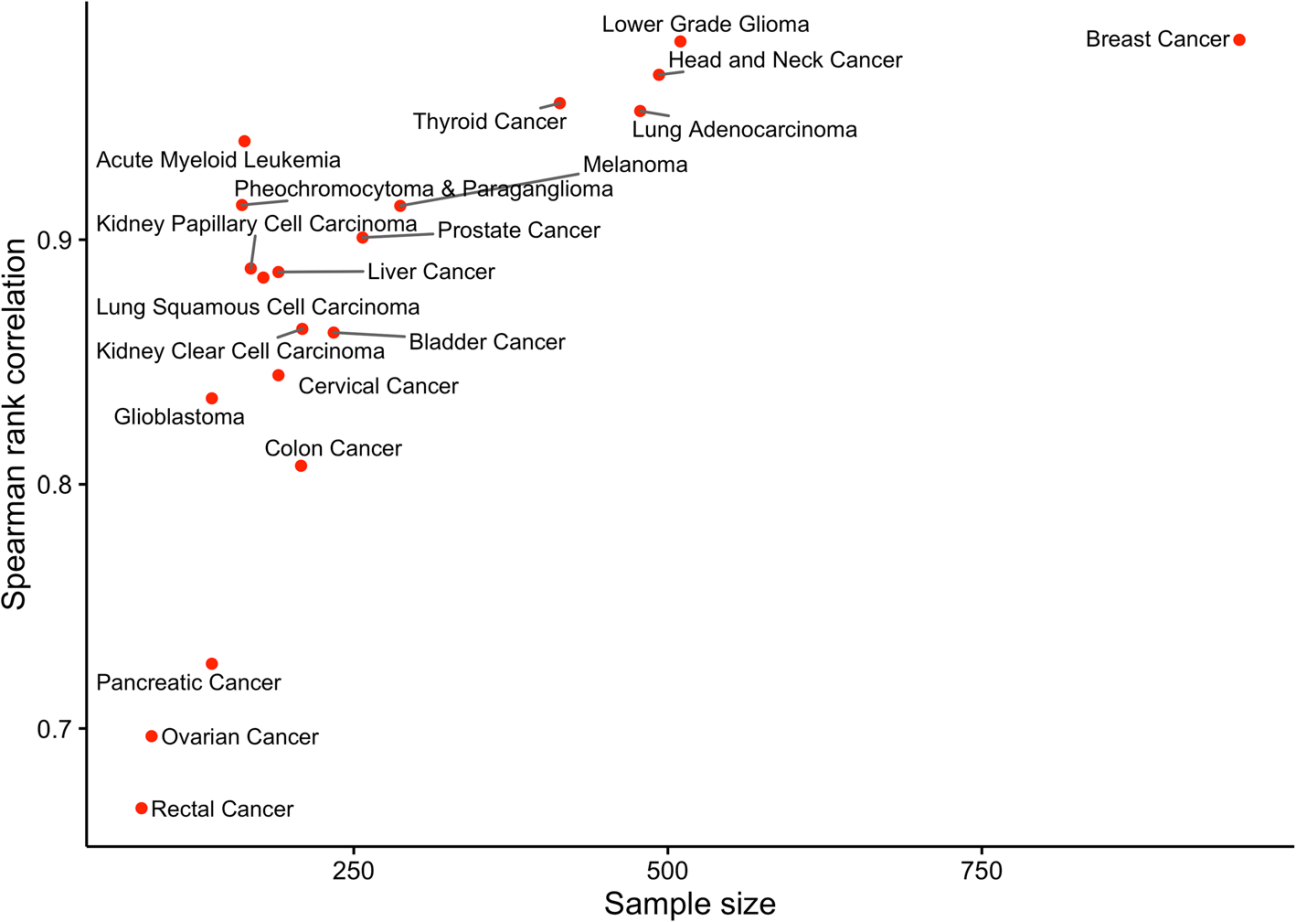
Correlation of $R^{2}_{pred}$ values between models fit using just COSMIC genes and models fit using all TCGA genes. Spearman rank correlation between $R^{2}_{pred}$ values for curated canonical pathway (C2.CP) models estimated using just COSMIC consensus cancer genes and the $R^{2}_{pred}$ values for C2.CP pathway models estimated using all TCGA genes. The rank correlation values for each TCGA cohort are plotted relative to cohort sample size

To determine if the models correctly capture cancer-specific phenomena, we compared the empirical distribution and rank ordering of $R^{2}_{pred}$ values obtained for pathway models fit using two different sets of predictors: 1) the somatic alteration status of genes in the COSMIC cancer gene census or 2) the somatic alteration status of all genes included in the TCGA data. As an expert curated list of mutated human genes that have an experimentally supported association with oncogenesis [29], the COSMIC cancer gene census provides a comprehensive list of known human cancer genes. We expected the empirical distribution of $R^{2}_{pred}$ values for these two potential predictor sets to be similar. Although the somatic alteration of non-cancer genes will impact gene expression levels within tumors, we expected that the ability to predict the variation of pathway activity across tumors of a single cancer type would be driven largely by the somatic alteration status of genes with a known cancer association. In other words, a model that included somatic alteration predictors for all TCGA genes would not have predictive performance significantly greater than a model that just included predictors for genes in the COSMIC cancer gene census. Following similar reasoning, we expected the rank ordering of pathways according to $R^{2}_{pred}$ to be to similar regardless of whether non-COSMIC genes were included as predictors. As seen in Fig. 3, the $R^{2}_{pred}$ empirical distribution is similar for both predictor sets with mean $R^{2}_{pred}$ values that are significantly larger than the mean generated using random data, matching our expectation. In fact, the mean $R^{2}_{pred}$ for models that include predictors for all TCGA genes is slightly lower than the mean $R^{2}_{pred}$ for models that use just COSMIC genes, confirming that the addition of non-COSMIC predictors does not meaningfully improve predictive performance. As seen in Fig. 4, the Spearman rank correlation between the $R^{2}_{pred}$ values computed using the two predictor sets increases with sample size and approaches 1 for the largest cohorts. These results are consistent with our expectation that pathway ranking depends primarily on somatic alterations in cancer-associated genes. The lower correlation values for the smaller cohorts reflects an expected increase in variance for the $R^{2}_{pred}$ estimates.

### Does model predictive performance identify biologically important pathways?

To address this question, we ranked the pathway models for each high-level TCGA cancer type according to $R^{2}_{pred}$ values. We expected that the pathways with the largest *R*^2^ values would represent biological processes that play an important role in oncogenesis for the analyzed cancer type, would be more likely to identify therapeutic targets and would have activity levels linked to patient prognosis. As an illustrative example, Table 1 lists the top MSigDB C2.CP pathways for the TCGA lung adenocarcinoma cohort (one of the larger cohorts with stable pathway ranking between predictor sets) with separate lists for the two potential predictor sets (i.e., somatic alterations for COSMIC concensus cancer genes or somatic alterations for all available TCGA genes). The Additional file 1 contains similar tables for the other four of the five largest TCGA cohorts (breast cancer, lower grade glioma, head and neck cancer and thyroid cancer) for both the C2.CP and C6 collections (see Additional file 1: Tables S3, S4, S6, S7, S9, S10, S15 and S16). As shown in Table 1, the top ten pathways for lung adenocarcinoma are almost identical for both potential predictor sets with a Spearman rank correlation for the entire C2.CP collection of 0.96. All of the top ten pathways for models built using just the COSMIC consensus cancer genes are related to the cell cycle, which has a well known association with lung adenocarinoma [32, 33]. Importantly, four of the top ten pathways (pathways with ranks 1, 2, 6 and 10) are associated with genes identified as either therapeutic targets for lung adenocarcinoma (Ran [34] and ATR [35, 36]) or as biomarkers of patient prognonsis (CDC6 [37]).

**Table 1:** Top pathway models for lung adenocarcinoma

Consensus cancer genes			All TCGA genes		
#	Gene set	$R^{2}_{pred}$	#	Gene set	$R^{2}_{pred}$
1	BIOCARTA_RANMS_PATHWAY	0.487	1	BIOCARTA_RANMS_PATHWAY	0.465
2	REACTOME_ACTIVATION_OF_ATR_IN_RESPO...	0.486	3	REACTOME_G2_M_CHECKPOINTS	0.459
3	REACTOME_G2_M_CHECKPOINTS	0.483	2	REACTOME_ACTIVATION_OF_ATR_IN_RESPO...	0.456
4	REACTOME_CELL_CYCLE	0.472	6	REACTOME_CDC6_ASSOCIATION_WITH_THE_...	0.450
5	REACTOME_MITOTIC_M_M_G1_PHASES	0.468	14	REACTOME_CHROMOSOME_MAINTENANCE	0.434
6	REACTOME_CDC6_ASSOCIATION_WITH_THE_...	0.468	9	REACTOME_G0_AND_EARLY_G1	0.433
7	REACTOME_DNA_REPLICATION	0.464	4	REACTOME_CELL_CYCLE	0.431
8	REACTOME_CELL_CYCLE_MITOTIC	0.464	8	REACTOME_CELL_CYCLE_MITOTIC	0.429
9	REACTOME_G0_AND_EARLY_G1	0.454	5	REACTOME_MITOTIC_M_M_G1_PHASES	0.428
10	PID_ATR_PATHWAY	0.450	7	REACTOME_DNA_REPLICATION	0.427

Top ten MSigDB C2.CP pathways ranked according to $R^{2}_{pred}$ for regression models constructed using the TCGA lung adenocarcinoma data. Separate rankings are shown for models estimated using both potential predictor sets (i.e., the somatic alterations for either genes in the COSMIC cancer gene census or all genes available in the TCGA data). The "#" columns contain the pathway rank according to the COSMIC models. Note that the complete names of the rank 2 and rank 6 pathways for COSMIC genes are REACTOME_ACTIVATION_OF_ATR_IN_RESPONSE_TO_REPLICATION_STRESS and REACTOME_CDC6_ASSOCIATION_WITH_THE_ORC_ORIGIN_COMPLEX

### Can the models be used to identify cancer driver genes?

To ascertain if the models can identify known cancer driver genes, we ranked the somatic alteration predictors for each cancer type according to predictor significance in the pathway models. Specifically, alterations were ranked according to a weight computed as the average across all pathway models in a specific MSigDB collection of the product of predictor significance (i.e., the -log(*p*-value) for the predictor in the unpenalized model) and model $R^{2}_{pred}$. An example of this ranking is shown in Table 2 for the TCGA lung adenocarcinoma cohort with ranks computed using all four combinations of MSigDB collection and predictor set (the Additional file 1 contains similar tables for the other four of the five largest TCGA cohorts, see Additional file 1: Tables S5, S8, S11, S17). It is important to note the potential bias in these weights caused by pathway overlaps. Specifically, the weights for somatic alterations associated with the expression of genes annotated to multiple pathways will be inflated relative to the weights for somatic alterations that are only associated infrequently annotated genes.

**Table 2:** Top predictors for lung adenocarcinoma

Consensus cancer genes						All TCGA genes					
C2.CP			C6			C2.CP			C6		
#	Predictor	W	#	Predictor	W	#	Predictor	W	#	Predictor	W
1	**TP53**	0.784	1	**TP53**	0.672	1	**KEAP1**	0.766	1	**KEAP1**	0.942
2	**SMARCA4**	0.559	3	MET (CNV)	0.590	2	**TP53**	0.484	2	**TP53**	0.423
3	MET (CNV)	0.445	2	**SMARCA4**	0.541	3	**SMARCA4**	0.339	4	**KRAS**	0.397
4	**KRAS**	0.374	4	**KRAS**	0.507	4	**KRAS**	0.249	3	**SMARCA4**	0.298
5	SETD2	0.268	8	**EGFR (CNV)**	0.319	5	**STK11**	0.171	8	*BAGE2	0.209
6	**RBM10**	0.263	25	**EGFR**	0.279	6	*DST	0.166	7	*FRG1B	0.148
7	**STK11**	0.239	9	FOXA1 (CNV)	0.262	7	*FRG1B	0.145	13	MET (CNV)	0.146
8	**EGFR (CNV)**	0.220	10	MYC (CNV)	0.241	8	*BAGE2	0.131	43	*PKHD1	0.137
9	FOXA1 (CNV)	0.213	44	EP300 (CNV)	0.229	9	*SPTA1	0.127	36	**EGFR**	0.130
10	MYC (CNV)	0.209	11	**SMARCA4 (CNV)**	0.227	10	*ANK2	0.115	6	*DST	0.127

Top ten gene-level somatic alteration predictors from models estimated using the TCGA lung adenocarcinoma cohort. The predictors are ranked according to a weight, W, computed as the average across all pathway models in the MSigDB collection of the product the -log(*p*-value) for the predictor in the unpenalized model and model $R^{2}_{pred}$. Separate rankings are shown for the C2.CP and C6 collections using both potential sets of predictors. The "#" columns contain the rank of the predictor in the list computed using C2.CP collection and the target predictor set. Predictors marked in bold represent known driver genes for lung adenocarcinoma. Predictors prefixed with a "*" are not in the COSMIC cancer gene census

**Fig. 5:**
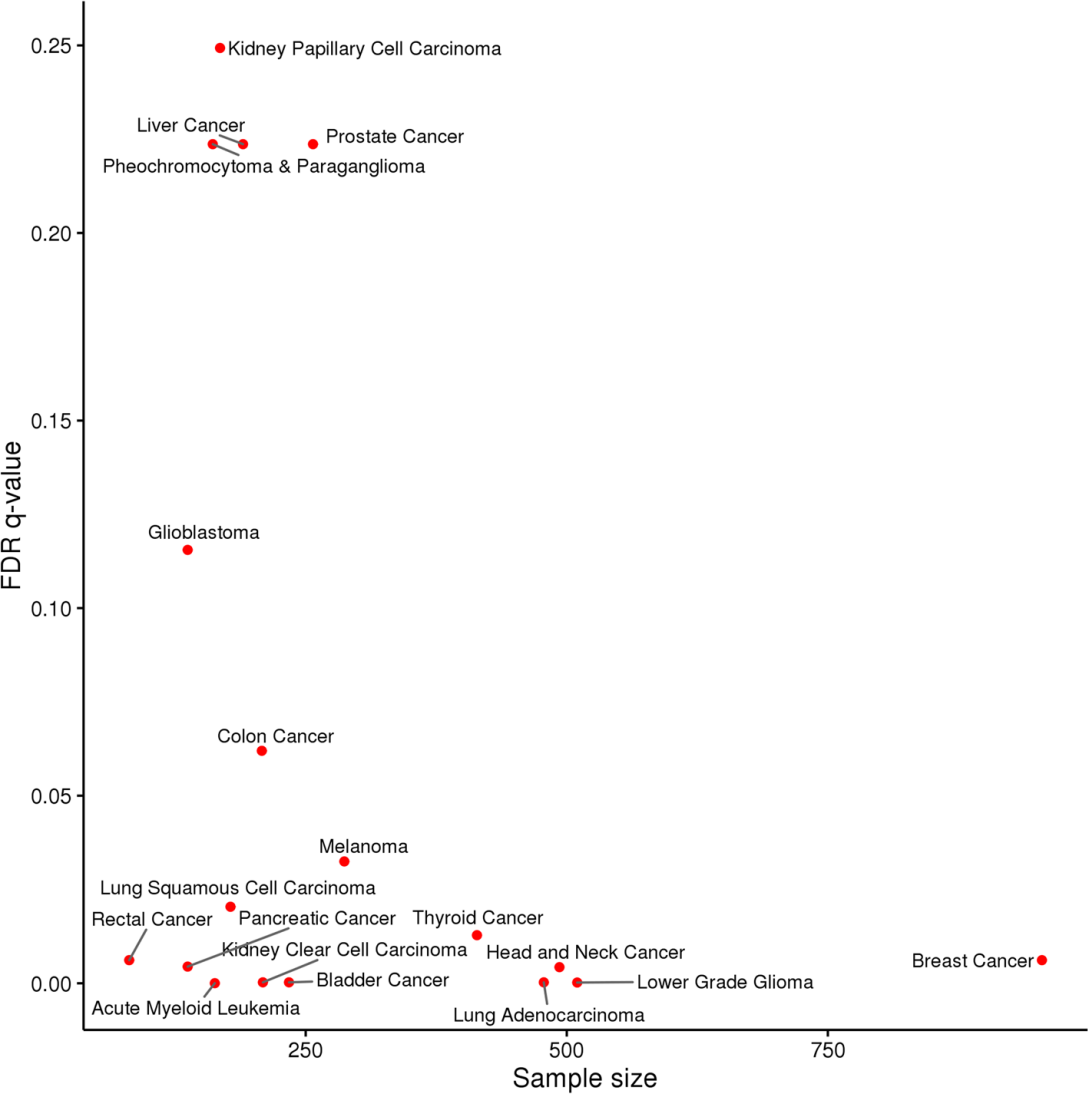
Enrichment of known cancer driver genes. The false discovery rate (FDR) q-values (as computed using the Benjamini and Hochberg (BH) [38] method) for the Wilcox rank sum tests of enrichment of known driver genes among the ranks of all predictors for the MSigDB C2.CP models using all TCGA genes. The q-value for each cancer type cohort is plotted relative to the cohort sample size. All but one cancer type has a q-value of < 0.1 with a general association between significance and sample size

We expected that the top ranking gene-level somatic alterations would disproportionately represent known driver genes. To evaluate this, we first generated a list of known driver genes for each cancer type using the cancer type associations from the COSMIC cancer gene census (see Additional file 1: Table S1 for details). Although not comprehensive, these represent cancer type relationships that are independent of the TCGA data (i.e., they are not inferred from TCGA somatic alteration data). Enrichment of these known driver genes among the ranked predictors associated with all available TCGA genes was then tested using a Wilcoxon rank sum test and false discovery rate (FDR) q-values were computed using the Benjamini and Hochberg (BH) [38] method. Consistent with our expectations, the enrichment q-values for all but three of the cancer types using the C2.CP models was below 0.07 with the majority below 0.01 (see Fig. 5 and S8). For the lung cancer example, a careful examination of the top 10 COSMIC predictors for the C2.CP collection (see Table 2) reveals that all ten in fact have a known association with lung adenocarcinoma (only six are in the list of known driver genes) with most also serving as therapeutic targets and/or prognostic indicators (TP53 [32], SMARCA4 [39], MET [40, 41], KRAS [42, 43], SETD2 [44], RBM10 [45], STK11 [46, 47], EGFR [43, 48], FOXA1 [49] and MYC [43]).

**Fig. 6:**
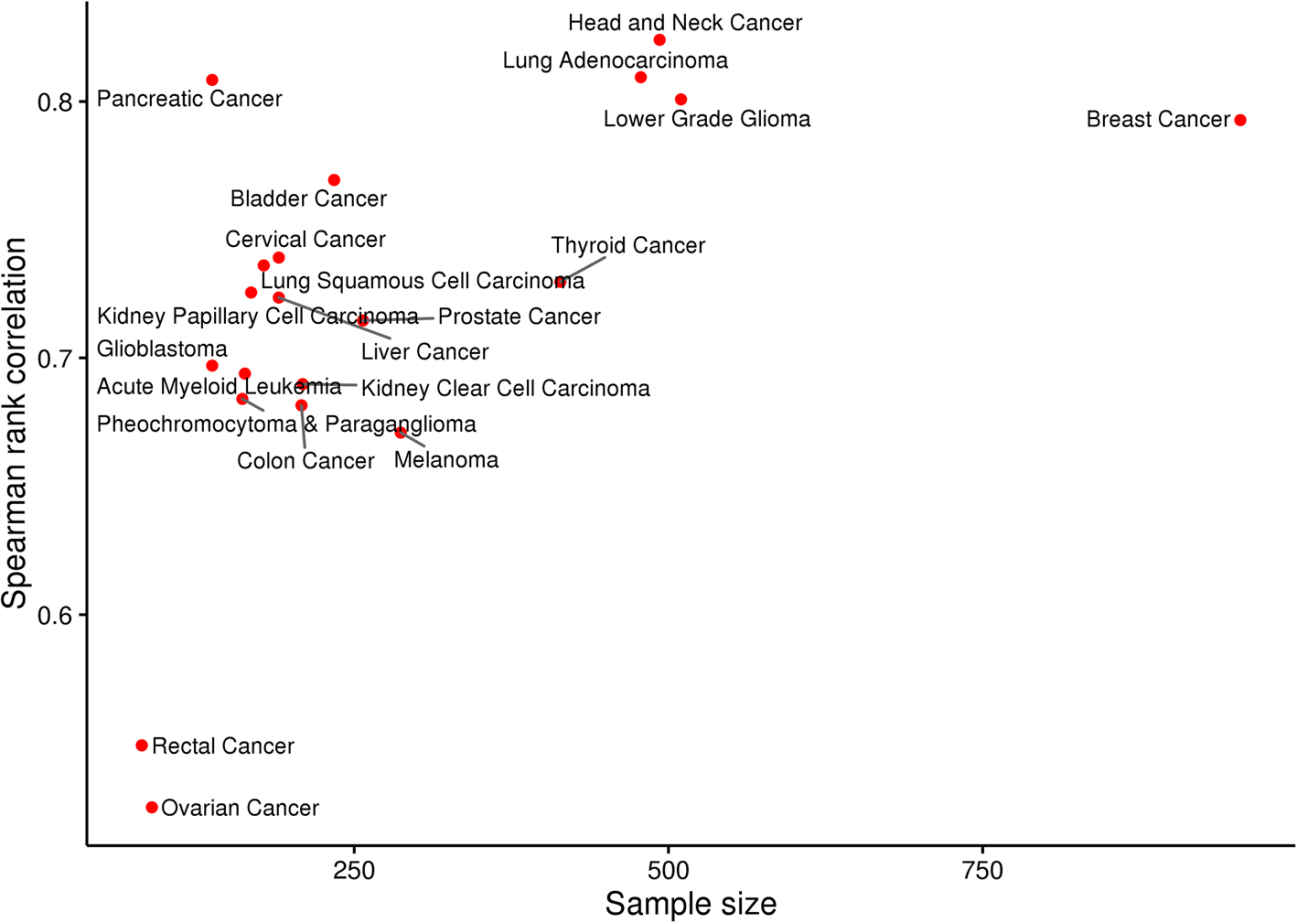
Correlation of predictor weights between C2.CP and C6 regression models. Spearman rank correlation between the weights for COSMIC consensus cancer gene predictors computed for the MSigDB curated canonical pathways (C2.CP) and oncogenic signatures (C6) collections. The correlation values for each TCGA cohort and are plotted relative to cohort sample size

We also expected that the predictor ranking would be insensitive to the pathway collection on which it was computed, i.e., the C2.CP and C6 rankings would be similar. As seen in Fig. 6, the Spearman rank correlation between the predictor weights computed using the C2.CP or C6 pathway models increases from ∼ 0.5 for the smallest cohorts to ∼ 0.8 for the largest cohorts. For the lung cancer example shown in Table 2, seven of the top ten predictors for the C2.CP collection are also in the top ten list for the C6 collection with this magnitude overlap holding for both predictor sets. These results are consistent with our expectation that predictor ranking is identifying true driver genes and provides additional evidence that pathways with large $R^{2}_{pred}$ values are associated with important aspects of cancer biology.

### Can the models be used to identify novel driver genes?

To determine if the models are effective at identifying novel driver genes, we examined the high-ranking somatic alteration predictors for genes not included in the COSMIC cancer gene census. In particular, we examined non-COSMIC predictors whose high-ranking was replicated across both the C2.CP and C6 collections. Because these genes do not have a well-established role in cancer but are significant predictors in models associated with different pathway collections, we hypothesized that they could represent novel drivers for the analyzed cancer type. For the lung adenocarcinoma cohort, the predictors prefixed with an * in the right two columns of Table 2 are associated with non-COSMIC genes. Importantly, three of the non-COSMIC predictors included in the top ten for the C2.CP collection (DST, FRG1B and BAGE2) are also in the top ten for the C6 collection and none of the three has an established association with lung adenocarcinoma. DST, FRG1B and BAGE2 are thus candidate driver genes for lung adenocarcinoma and good targets for follow-on experiments.

### Is model predictive performance sufficient for personalized pathway analysis?

The $R^{2}_{pred}$ values computed via CV on the TCGA data sets reflect the predictive performance that can be expected for personalized pathway analysis. Although more research is needed to determine the true utility of these models for personalized pathway analysis, we believe that the current results are encouraging, especially for the larger TCGA cohorts and pathways models that have $R^{2}_{pred}$ values around ∼ 0.5. These models, when combined with model uncertainty to generate a prediction interval, may provide useful information regarding pathway dysregulation within a single tumor. The prediction of expression-based activity from somatic alteration status may be especially useful for cases where gene expression data is unavailable. Appropriately, this approach to personalized pathway analysis is strongly influenced by somatic alterations of common cancer driver genes, i.e., the somatic alteration of a gene like p53 will impact the predicted aberrant activity of many pathways whose expression-based activity is closely associated with somatic alterations in the tumor. This follows from the criteria used by our method to identify candidate driver genes, i.e., the somatic alteration predictors that are significant in highly predictive regression models are assumed to represent potential cancer driver genes. Improvements in predictive performance of this approach can likely be achieved with larger sample sizes (see Additional file 1: Figures S4 and S5 for the association between mean $R^{2}_{pred}$ and cohort size) or with the inclusion of additional predictors such as methylation values.

### Can the models be used to characterize cancer subtypes?

Although the TCGA cohorts provide a useful high-level grouping of cancers, significant heterogeneity often exists within each type of cancer. For example. breast cancers are often sub-divided according to gene expression or mutational profiles [50] into two (e.g., luminal-like and basal-like) or more (e.g., luminal A, luminal B, HER2 enriched, basal-like) subtypes. To determine if the pathway models fit for the high-level TCGA cancer types could be used to characterize the features of cancer subtypes, we explored the differences in predicted pathway activity and somatic mutation predictors for the TCGA BRCA subjects assigned to either the basal or luminal PANCAN cluster-of-cluster assignments [51] (see Section 1.4 of the Additional file 1 for analysis details). As illustrated in the Additional file 1 (Tables S18-S23), the differences in predicated pathway activity and important somatic alterations are consistent with known differences between luminal and basal breast cancer types subtypes.

## Discussion

We have described a novel approach for jointly analyzing gene expression and somatic alteration data through the lens of biological pathways. Our approach combines single sample pathway analysis on gene expression data with LASSO-penalized regression to build statistical models that use the somatic alterations present in each tumor to predict the deviation of pathway activity from the expected activity for the associated cancer type. Because this method can analyze case-only data and does not require information regarding pathway topology, it allows researchers to explore data sets that cannot be analyzed by existing cancer pathway analysis techniques. These models can be used to achieve our three primary aims:

*Aim 1: Identify pathways that play an important role in the pathophysiology of human cancers.* By ranking the pathway-specific regression models according to predictive performance, it is possible identify pathways whose activity is driven by somatic alterations in the tumor. These pathways can be expected to play a key role in the pathophysiology of each cancer type and are thus strong candidates for therapeutic intervention. As shown through our analysis of TCGA data using MSigDB pathways, the regression models are capturing real cancer-specific phenomena. A qualitative analysis of the models with the largest predictive performance indicates that the associated pathways have a clear relationship with the cancer type and, in many cases, identify therapeutic targets.

*Aim 2: Identify genes whose somatic alteration is significantly associated with pathway activity.* By ranking the gene-level somatic alteration predictors according to predictor significance and model predictive performance, it is possible to identify the genes whose somatic alteration drives pathway activity for each cancer type. As demonstrated by the analysis of TCGA data and MSigDB pathways, the predictor rankings can be replicated across disjoint pathway collections and are significantly enriched for genes known to be associated with each cancer type. By fitting regression models using somatic alterations for genes without a known cancer association, our approach also supports the discovery of novel driver genes.

*Aim 3: Support personalized pathway analysis using only somatic alteration data.* The predictive performance estimated for the MSigDB pathway models on the TCGA data reflects the expected performance for personalized pathway analysis. Although additional software engineering will be required to create a tool that can be easily used by other researchers for personalized pathway analysis, the current results are promising and motivate future work in this direction. For cases in which gene expression data is unavailable and somatic alteration data may be limited to known cancer driver genes, these models can provide useful information on the activity of pathways within a patient’s tumor; information that may help assess prognosis or guide treatment.

### Limitations

Important limitations of our method and the reported results include the scope and quality of the data drawn from the TCGA, COSMIC and MSigDB, limitations of the statistical models and estimation approaches employed for pathway analysis, and limitations associated with the subjective interpretation of pathways associated with highly predictive regression models. Limitations associated with the TCGA data include the small number of samples for many of the analyzed cohorts, the heterogeneity of tumors within each cohort (e.g., the major subtypes of breast cancer), errors in the gene-level estimates of non-silent somatic mutations and copy number variation, sparsity of the somatic alteration data, and the fact that the employed non-silent mutation indicators fail to distinguish between gain-of-function and loss-of-function mutations. An additional limitation associated with the leveraged TCGA data is the fact that somatic alteration data types like methylation, mutations of non-protein coding genes and structure features such as fusions and translations are not included as predictors in the regression models. Although the COSMIC cancer gene census provides a comprehensive list of genes with a known cancer association, the census does not quantify the degree or direction of association, provides only approximate cancer type associations for each gene, and likely misses many genes that have a true link to cancer. Limitations of the gene set collections in MSigDB include the variable quality of annotations, bias in pathway annotations (i.e., more annotations will exist for well studied genes and pathways), the fact that the analyzed MSigDB pathways do not represent all potential cancer-related pathways, and overlaps between the members of many pathways. An important implication of our use of curated pathways from MSigDB is that our method is unlikely to identify truly novel pathway-cancer associations. The statistical model used to predict pathway activity only includes copy number alterations and indicators of non-silent somatic alterations as predictors; other genomic features that are known to impact gene expression such as epigenetic changes (e.g., methylation), translocations, gene fusions and mutation of non-protein coding genes are ignored. Given the unknown marginal and joint distribution of the $R^{2}_{pred}$ values, a formal statistical test was not performed on individual $R^{2}_{pred}$ values or comparing the different $R^{2}_{pred}$ distributions shown in Fig. 3. Other analytical limitations include the fact that the scores generated by GSVA only approximate pathway activity within each tumor, and the stochastic nature of LASSO-penalized estimation. An important limitation of the evaluation results is the qualitative and subjective analysis used to ascertain the cancer relevance and therapeutic value of identified pathways and genes. While the estimated regression models provide useful insight into the somatic alterations can drive pathway dysregulation in the analyzed TCGA cancer types, model performance has not been evaluated on non-TCGA data and the current models and implementation logic do not support the direct use of these models for personalized pathway analysis on new patient tumor data.

### Future directions

Possible extensions to this method include support for additional data types, modifications to the statistical model, exploration of cancer subtypes, validation on other cancer genomics data sets and creation of tools to support personalized pathway analysis on new tumor genomic data. To more accurately model the somatic alterations that drive gene expression, the current approach can be expanded to include epigenetic modifications (e.g., methylation), mutations of non-protein coding genes, and features such as gene fusions and translocations as predictors in the regression model. An important issue that must be address in future efforts to integration additional predictor variables will be the limited available of many of these additional genomic data types. Potential enhancements of the statistical model include the addition of interaction terms, predictor weighting based on prior knowledge regarding the role of specific genes in cancer and modification of predictor weights to account for the overlap between pathways. To support formal statistical analysis of the $R^{2}_{pred}$ values computed for each pathway model, resampling approaches could be used (i.e., generate multiple bootstrap resampled versions of the TCGA data and estimate pathway regression models for each resampled data set). Alternative approaches for computing the single sample pathway scores can also be investigated, e.g., generate GSVA statistics that can identify gene sets with both up and down-regulated members, base single sample scores on gene expression relative to controls or another cancer type, etc. Evaluation of this approach can be expanded to include the analysis of other cancer types or subtypes of the analyzed cohorts (e.g., extend the analysis of breast cancer subtypes to other cancers), and the analysis of data from other cancer genomics repositories. A particularly important topic for future research involves the use of more a objective and systematic approach for evaluating identified pathways and genes with experimental confirmation of any novel findings.

## Conclusions

We have developed a new approach for the pathway-based analysis of multi-omics cancer data. Our approach combines single-sample pathway analysis with multi-stage, lasso-penalized regression to find pathways whose gene expression can be explained largely in terms of the non-silent somatic mutations and copy number variations present in the tumor. This method enables the identification of biologically important pathways and genes and can be used for personalized pathway analysis in cases where gene expression data is unavailable. Importantly, this method can be used on case-only data sets and does not require information regarding pathway topology. An analysis of 20 human cancer types using TCGA genomic data and MSigDB gene sets illustrates the effectiveness of our technique. These analysis results also provide cancer researchers with ranked lists of pathways and genes that likely play a key role in the etiology of these cancer types, information that can be used to generate hypotheses for more detailed experimental exploration of cancer pathways and novel driver genes.

## Additional file

Additional file 1 Additional results and details on the computational pipeline, analyzed data sets and logic used to generate all tables and figures. (PDF 1772 kb)

## Abbreviations

C2.CP: MSigDB curated canonical pathways collection
C6: MSigDB oncogenic signatures collection
CNV: Copy number variation
COSMIC: Catalog of somatic mutations in cancer
GO: Gene ontology
GSVA: Gene set variation analysis
LASSO: Least absolute shrinkage and selection operator
MSigDB: Molecular signatures database
TCGA: The cancer genome atlas
