# Supplementary material for: A multi-omics approach for identifying important pathways and genes in human cancer
Source: BMC Bioinformatics. 2018 Dec 12;19:479. doi: 10.1186/s12859-018-2476-8 (PMC6292115; doi:10.1186/s12859-018-2476-8)
Supplement: Supplementary file 1 — Additional results and details on the computational pipeline, analyzed data sets and logic used to generate all tables and figures. (PDF 1772 kb) [file 12859_2018_2476_MOESM1_ESM.pdf]

Supplemental Material: A multi-omics approach for identifying important pathways and genes in human cancer

H. Robert Frost and Christopher I. Amos

Contents

1 Supplemental Methods **2**
1.1 Pathway Definitions . . . 2
1.1.1 Cancer Driver Genes . . . 3
1.2 Tumor Genomic Data . . . 3
1.3 Analysis pipeline . . . 6
1.4 Analysis of breast cancer subtypes . . . 8
1.5 Generation of tables and figures . . . 9

2 Supplemental Results **11**

List of Tables

S1 Driver genes for 20 TCGA pan cancer cohorts used in the analysis as defined by the COSMIC cancer gene census. . . . 4
S2 Sample and gene counts for the 20 TCGA pan cancer cohorts used in the analysis. Note that for the number of mutation and CNV predictors, the same gene may be counted twice (i.e., both non-silent somatic mutation data and CNV data is available for the gene). . . . 5
S3 Top MSigDB C2.CP pathway models for Breast Cancer . . . 16
S4 Top MSigDB C6 pathway models for Breast Cancer . . . 16
S5 Top predictors for Breast Cancer . . . 16
S6 Top MSigDB C2.CP pathway models for Head and Neck Cancer . . . 17
S7 Top MSigDB C6 pathway models for Head and Neck Cancer . . . 17
S8 Top predictors for Head and Neck Cancer . . . 17
S9 Top MSigDB C2.CP pathway models for Lower Grade Glioma . . . 18
S10 Top MSigDB C6 pathway models for Lower Grade Glioma . . . 18
S11 Top predictors for Lower Grade Glioma . . . 18
S12 Top MSigDB C2.CP pathway models for Lung Adenocarcinoma . . . 19
S13 Top MSigDB C6 pathway models for Lung Adenocarcinoma . . . 19
S14 Top predictors for Lung Adenocarcinoma . . . 19
S15 Top MSigDB C2.CP pathway models for Thyroid Cancer . . . 20
S16 Top MSigDB C6 pathway models for Thyroid Cancer . . . 20
S17 Top predictors for Thyroid Cancer . . . 20
S18 MSigDB C2.CP pathways differentially active in luminal vs. basal breast cancer . . . 21
S19 MSigDB C6 pathways differentially active in luminal vs. basal breast cancer . . . 21
S20 Genes whose somatic mutation drives dysregulation of MSigDB C2.CP pathways. Relative importance in luminal vs. basal breast cancer. . . . 21
S21 Genes whose somatic mutation drives dysregulation of MSigDB C6 pathways. Relative importance in luminal vs. basal breast cancer. . . . 21
S22 Genes whose copy number variation drives dysregulation of MSigDB C2.CP pathways. Relative importance in luminal vs. basal breast cancer. . . . 22
S23 Genes whose copy number variation drives dysregulation of MSigDB C6 pathways. Relative importance in luminal vs. basal breast cancer. . . . 22
S24 Top predictors for Pancreatic Cancer using GSVA . . . 23

S25 Top predictors for Pancreatic Cancer using ssGSEA 23

List of Figures

S1 Illustration of results from the analysis of TCGA pan cancer data using the MSigDB oncogenic signatures (C6) collection. Rows correspond to the TCGA cohorts and columns to MSigDB gene sets. Each cell represents a pathway and cancer type-specific regression model colored according to the R^2_{pred} value. Both the columns and rows are ordered according to the output from hierarchical agglomerative clustering using complete link agglomeration and euclidean distance. 11
S2 Box plot illustrating the distribution of R^2_{pred} values from the analysis of TCGA pan cancer data using the MSigDB oncogenic signatures (C6) collection. Values are shown for three different analyses: *COSMIC*: estimation of pathway regression models using somatic alteration data for COSMIC consensus cancer genes, *All TCGA*: estimation of models using somatic alteration data for all genes included in the TCGA datasets, and *Random*: estimation of models using randomized somatic alteration data for COSMIC consensus cancer genes. 12
S3 Spearman rank correlation between R^2_{pred} values for C6 pathway models estimated using just COSMIC consensus cancer genes and the R^2_{pred} values for C2.CP pathway models estimated using all TCGA genes. The rank correlation values are computed separately for each TCGA cohort and are plotted relative to cohort sample size. 12
S4 Mean R^2_{pred} values for C2.CP pathway models estimated for each TCGA cohort using just COSMIC consensus cancer genes. The mean R^2_{pred} values are plotted relative to cohort sample size. 13
S5 Mean R^2_{pred} values for C6 pathway models estimated for each TCGA cohort using just COSMIC consensus cancer genes. The mean R^2_{pred} values are plotted relative to cohort sample size. 13
S6 Spearman rank correlation between the weights for somatic alteration predictors for all available TCGA genes computed for the MSigDB C2.CP and C6 collections. The rank correlation values are computed separately for each TCGA cohort and are plotted relative to cohort sample size. 14
S7 The false discovery rate (FDR) q-values (as computed using the Benjamini and Hochberg (BH) (Benjamini and Hochberg, 1995) method) for the Wilcox rank sum tests of enrichment of known driver genes among the ranks of all predictors for the MSigDB C6 models using all TCGA genes. The q-value for each cancer type cohort is plotted relative to the cohort sample size. 15

1 Supplemental Methods

1.1 Pathway Definitions

We assume there are p_a genes grouped into m overlapping gene sets or pathways as defined by an $m \times p_a$ gene set indicator matrix $\mathbf{A}$:

$$\mathbf{A} = \begin{bmatrix} a_{1,1} & \cdots & a_{1,p_a} \\ \vdots & \ddots & \vdots \\ a_{m,1} & \cdots & a_{m,p_a} \end{bmatrix} \tag{1}$$

where $a_{i,j}$ is 1 if gene j belongs to gene set i and 0 otherwise.

For the reported analyses, the $\mathbf{A}$ matrix was populated using gene sets from version 5.2 of the Molecular Signatures Database (MSigDB) (Liberzon *et al.*, 2011). Specifically, the MSigDB C2.CP (curated canonical pathways) and C6 (oncogenic signatures) collections were used. Let the $\mathbf{A}$ matrices for the C2.CP and C6 collections be represented as $\mathbf{A_{C2.CP}}$ and $\mathbf{A_{C6}}$.

- C2.CP
  - Number of gene sets: 1329
  - Number of unique genes in all sets: 8,904
  - MSigDB description: "Gene sets from the pathway databases. Usually, these gene sets are canonical representations of a biological process compiled by domain experts."
  - Pathways are drawn from 9 different databases:
    * BioCarta (http://www.genecarta.com)

* KEGG (http://www.genome.jp/kegg)
* Matrisome (http://matrisomeproject.mit.edu)
* Pathway Interaction Database (http://pid.nci.nih.gov)
* Reactome (http://www.reactome.org)
* SigmaAldrich (http://www.sigmaaldrich.com/life-science.html)
* Signaling Gateway (http://www.signaling-gateway.org)
* Signal Transduction KE (http://stke.sciencemag.org)
* SuperArray (http://www.superarray.com)

- C6
  - Number of gene sets: 189
  - Number of unique genes in all sets: 11,250
  - MSigDB description: "Gene sets represent signatures of cellular pathways which are often dis-regulated in cancer. The majority of signatures were generated directly from microarray data from NCBI GEO or from internal unpublished profiling experiments which involved perturbation of known cancer genes. In addition, a small number of oncogenic signatures were curated from scientific publications."

1.1.1 Cancer Driver Genes

We assume that information on the known associations between p_d genes (where $p_d \geq p_a$) and t cancer types is defined by a $t \times p_d$ matrix $\mathbf{D}$ of indicator variables:

$$\mathbf{D} = \begin{bmatrix} d_{1,1} & \cdots & d_{1,p_d} \\ \vdots & \ddots & \vdots \\ d_{t,1} & \cdots & d_{t,p_d} \end{bmatrix} \tag{2}$$

where $d_{i,j}$ is 1 if gene j has a known association with cancer type i. Because the set of t cancer types is not comprehensive and type-specific associations may be only approximate or unknown for some genes, we also assume that the overall cancer association for each gene is held in a separate vector of indicator variables:

$$\mathbf{o} = (o_1, ..., o_{p_d}) \tag{3}$$

where o_i is 1 if gene i has a known association with any cancer type (not necessarily one of the t types defined in $\mathbf{D}$). Let the total number of cancer-associated genes be represented by b:

$$b = \sum_{i=1}^{p_d} o_i \tag{4}$$

For the reported analyses, the $\mathbf{D}$ matrix and $\mathbf{o}$ vector were generated using the cancer type annotations in the COSMIC cancer gene census (release v78, 5th September 2016) (Forbes *et al.*, 2015):

- Contains cancer associations for 602 genes, i.e., b, as defined in (4), equals 602.
- COSMIC description: "The cancer Gene Census is an ongoing effort to catalogue those genes for which mutations have been causally implicated in cancer."

Table S1 lists the driver genes for the 20 TCGA cohorts used in the analysis (see next section for more details on the 20 TCGA cohorts). These lists were computed via string matching on the cancer type annotations in the COSMIC cancer gene census.

1.2 Tumor Genomic Data

We assume that gene expression and gene-level somatic alteration data has been collected on tumor samples for t different cancer types. Let the number of tumors with expression and somatic alteration data for each of the cancer types be represented by the vector $\{n_1, ..., n_t\}$. In particular, we assume existence of the following types of tumor genomic data for each cancer type $k, k \in 1...t$:

Cohort	# Drivers	Names
Acute Myeloid Leukemia	91	ABI1, ABL2, ACSL6, ARHGAP26, ARHGEF12, ARNT, BCOR, BCORL1, BCR, BRIP1, CASC5, CBFA2T3, CBFB, CBL, CBLB, CBLC, CDX2, CEBPA, CHIC2, CLP1, CREBBP, CSF3R, CUX1, DDX10, DEK, DNMT3A, ELF4, EP300, ERG, FANCA, FANCC, FANCD2, FANCE, FANCF, FANCG, FLT3, FNBP1, FUS, GAS7, GATA2, GMPS, HOXA13, HOXA9, HOXC11, HOXC13, HOXD11, HOXD13, JAK2, KAT6A, KAT6B, KDM5A, KIT, KMT2A, KRAS, LASP1, LEF1, MECOM, MLF1, MN1, MNX1, MYH11, NCOA2, NPM1, NRAS, NSD1, NUP214, NUP98, PALB2, PDGFRB, PER1, PICALM, PRDM16, PRRX1, PSIP1, PTPN11, RAD21, RPL22, RPN1, RUNX1, RUNX1T1, SBDS, SEPT5, SEPT6, SEPT9, SET, SETBP1, SH2B3, TET1, TOP1, TRIP11, WHSC1L1
Bladder Cancer	12	DROSHA, ERBB3, FGFR3, HRAS, KDM6A, MDM4, NOTCH1, NOTCH2, STAG2, TBL1XR1, TERT, TSC1
Breast Cancer	34	AKT1, APOBEC3B, ARID1A, ARID1B, BAP1, BRCA1, BRCA2, BRIP1, CASP8, CCND1, CDH1, CDKN1B, CHEK2, CTCF, EP300, ERBB2, ESR1, ETV6, FOXA1, GATA3, KEAP1, MAP2K4, MAP3K1, MAP3K13, NCOR1, NOTCH1, NTRK3, PALB2, PBRM1, PIK3CA, RB1, SMARCD1, TBX3, TP53
Cervical Cancer	1	MAPK1
Colon Cancer	42	AKT1, APC, AXIN1, AXIN2, B2M, BRAF, C2orf44, CTNNB1, CUX1, EIF3E, EP300, ERBB3, FBXW7, GRIN2A, HIF1A, KRAS, MAP2K1, MAP2K4, MAX, MDM2, MLH1, MSH2, MSH6, MUTYH, PIK3CA, PIK3R1, PMS1, PMS2, POLE, PTPRK, RAD21, RSPO2, RSPO3, SMAD2, SMAD3, SMAD4, TBL1XR1, TCF7L2, TGFBR2, TP53, UBR5, VTI1A
Glioblastoma	13	DAXX, GOPC, HIF1A, IDH1, IDH2, LZTR1, MDM4, PDGFRA, PIK3CA, PIK3R1, ROS1, STAG2, TERT
Head and Neck Cancer	11	BCORL1, CTCF, ERBB3, FAT1, FAT4, MTOR, NFE2L2, NOTCH1, PTK6, TGFBR2, TSC2
Kidney Clear Cell Carcinoma	29	ARID1A, ATP1A1, ATP2B3, BAP1, CACNA1D, CLTC, FH, FLCN, HIF1A, KCNJ5, KDM5C, KDM6A, KMT2D, MALAT1, MET, MTOR, NF2, NONO, PBRM1, PRCC, PRKACA, PTK6, SETD2, SFPQ, TFE3, TFEB, TSC1, TSC2, VHL
Kidney Papillary Cell Carcinoma	29	””
Liver Cancer	16	APC, ARID1B, ARID2, AXIN1, AXIN2, CASP8, CTNNB1, DNAJB1, FAT4, HNF1A, IL6ST, PRKACA, PTPN13, SMAD2, SMAD3, TERT
Lower Grade Glioma	20	APC, ATM, CDKN2C, CIC, EGFR, FUBP1, H3F3A, HIST1H3B, KLF6, MDM2, NBN, NF1, PMS2, PTEN, SDHA, SDHAF2, SDHB, SDHC, SDHD, TP53
Lung Adenocarcinoma	46	AKT1, ALK, BAP1, BRAF, CCDC6, CD74, DDR2, DROSHA, EGFR, EML4, ERBB2, ERBB4, EZR, FGFR2, GRIN2A, HIF1A, HIP1, KDR, KEAP1, KIF5B, KRAS, LRIG3, MALAT1, MAP2K1, MAP2K2, MYCL, NFE2L2, NKX2-1, NOTCH1, NRG1, PTPN13, RAD21, RB1, RBM10, RET, ROS1, SDC4, SLC34A2, SMARCA4, SOX2, STK11, STRN, TFG, TP53, TPM3, TPR
Lung Squamous Cell Carcinoma	46	””
Melanoma	31	ATF1, B2M, BAP1, BRAF, CDK4, CDKN2A, CDKN2A(p14), CUX1, DDB2, ERBB4, ERCC2, ERCC3, ERCC4, ERCC5, FAT4, GNA11, GNAQ, GRIN2A, KIT, MAP2K1, MAP2K2, MITF, NFKBIE, NRAS, PPP6C, RAC1, STAG2, TERT, TRRAP, XPA, XPC
Ovarian Cancer	27	AKT1, AKT2, ARID1A, ATR, BRAF, BRCA1, BRCA2, CCNE1, CDK12, CTNNB1, ERBB2, FOXL2, GOPC, LRP1B, MAPK1, MLH1, MSH2, MSH6, PIK3R1, PMS1, PMS2, PPP2R1A, PTK6, RNF43, ROS1, SMARCA4, STK11
Pancreatic Cancer	19	AKT2, APC, ATRX, BRAF, BRCA2, CDKN2A, CDKN2A(p14), DAXX, EP300, FAT1, FAT4, HIF1A, KRAS, MAP2K4, MEN1, RNF43, SMAD4, SND1, STK11
Pheochromocytoma & Paraganglioma	8	MAX, RET, SDHA, SDHAF2, SDHB, SDHC, SDHD, VHL
Prostate Cancer	25	ACSL3, AR, AXIN1, BRAF, CANT1, DDX5, ELK4, ERG, ETV1, ETV4, ETV5, FOXA1, HERPUD1, HMGN2P46, HNRNPA2B1, KLF6, KLK2, NCOR2, NDRG1, PTEN, RAF1, SLC45A3, SPOP, TMPRSS2, ZFHX3
Rectal Cancer	39	AKT1, APC, AXIN1, AXIN2, B2M, BRAF, C2orf44, CTNNB1, CUX1, EIF3E, EP300, FBXW7, GRIN2A, KRAS, MAP2K1, MAP2K4, MDM2, MLH1, MSH2, MSH6, MUTYH, PIK3CA, PIK3R1, PMS1, PMS2, POLE, PTPRK, RAD21, RSPO2, RSPO3, SMAD2, SMAD3, SMAD4, TBL1XR1, TCF7L2, TGFBR2, TP53, UBR5, VTI1A
Thyroid Cancer:	30	AKAP9, BRAF, CCDC6, CDC73, CDKN1B, DICER1, ERC1, GOLGA5, HMGA1, HOOK3, KRAS, KTN1, MEN1, MTOR, NCOA4, NRAS, NTRK1, PAX8, PCM1, PPARG, PRKAR1A, RET, STRN, TFG, TPM3, TPR, TRIM27, TRIM33, TSHR, ZNF331

Table S1: Driver genes for 20 TCGA pan cancer cohorts used in the analysis as defined by the COSMIC cancer gene census.

- Normalized gene expression data (either microarray gene expression of RNA-seq) measured on the tumor samples associated with cancer type k. Let the number of the p_a genes captured in either $\mathbf{A_{C2.CP}}$ or $\mathbf{A_{C6}}$ that have non-constant gene expression data for all n_k subjects be represented by pe_k. This gene expression data is for defined by an $n_k \times pe_k$ matrix $\mathbf{E_k}$:

$$\mathbf{E_k} = \begin{bmatrix} e_{1,1} & \cdots & e_{1,pe_k} \\ \vdots & \ddots & \vdots \\ e_{n_k,1} & \cdots & e_{n_k,pe_k} \end{bmatrix} \quad (5)$$

  where $e_{i,j}$ represents the abundance of the mRNA associated with gene j for sample i from cancer type k.

- Data on non-silent somatic mutations in the protein coding region of genes in tumor samples associated with cancer type k. Let the number of genes with non-constant somatic mutation indicators for all n_k subjects be

represented by pm_k, which can take on two separate values depending on whether the somatic mutations are limited to just the b genes with a cancer association as defined in (4) or include all genes with somatic mutation data in the data set. This somatic mutation data is recorded as gene-level mutation indicators in an $n_k \times pm_k$ matrix $\mathbf{M_k}$:

$$\mathbf{M_k} = \begin{bmatrix} m_{1,1} & \cdots & m_{1,pm_k} \\ \vdots & \ddots & \vdots \\ m_{n_k,1} & \cdots & m_{n_k,pm_k} \end{bmatrix} \tag{6}$$

where $m_{i,j}$ is 1 if there is at least one non-silent mutation in the protein coding region of the gene j for sample i from cancer type k.

- Copy number variation (CNV) data for genes in tumor samples associated with cancer type k. Let the number of genes with non-constant CNV values for all n_k subjects be represented by pc_k. Similar to pm_k, pc_k can take on two separate values depending on whether the CNV values are limited to just the b genes with a cancer association as defined in (4) or include all genes with CNV data in the data set. This CNV data is recorded as gene-level values in an $n_k \times pc_k$ matrix $\mathbf{C_k}$:

$$\mathbf{C_k} = \begin{bmatrix} c_{1,1} & \cdots & c_{1,pc_k} \\ \vdots & \ddots & \vdots \\ c_{n_k,1} & \cdots & c_{n_k,pc_k} \end{bmatrix} \tag{7}$$

where $c_{i,j}$ is an estimate of the copy number for gene j and sample i from cancer type k.

For the reported analyses, the $\mathbf{E_k}$, $\mathbf{M_k}$ and $\mathbf{C_k}$ matrices were populated using data from The Cancer Genome Atlas (TCGA) (Cancer Genome Atlas Research Network *et al.*, 2013) as downloaded from the UCSC Cancer Browser (Goldman *et al.*, 2015) pan cancer data set for the 20 TCGA cohorts that had the most samples with expression, mutation and CNV data (i.e., n_k). Table S2 lists these cohorts along with sample size details for the different data types. For all cohorts, the number of MSigDB collection genes (i.e., the columns of $\mathbf{A_{C2.CP}}$ or $\mathbf{A_{C6}}$) that had gene expression data for all n_k subjects (i.e., pe_k) was 8,427 for C2.CP (out of 8,904 unique C2.CP genes) and 10,618 for C6 (out of 11,250 unique C6 genes).

Cohort	Total samples	Samples with RNA-seq, mut. and CNV data (n_k)	Mut. and CNV predictors for all n_k ($pm_k + pc_k$) (Just COSMIC / all TCGA)
Acute Myeloid Leukemia	200	163	666/26,084
Bladder Cancer	432	234	1,103/40,692
Breast Cancer	1216	955	1,090/41,443
Cervical Cancer	313	190	1,039/39,479
Colon Cancer	504	208	1,099/40,825
Glioblastoma	607	137	783/30,001
Head and Neck Cancer	574	493	1,107/41,607
Kidney Clear Cell Carcinoma	605	209	952/35,380
Kidney Papillary Cell Carcinoma	326	168	852/31,858
Liver Cancer	429	190	986/35,350
Lower Grade Glioma	530	510	1,048/37,782
Lung Adenocarcinoma	662	478	1,129/42,859
Lung Squamous Cell Carcinoma	564	178	1,064/39,372
Melanoma	480	287	1,136/42,842
Ovarian Cancer	594	89	732/29,095
Pancreatic Cancer	190	137	1,015/37,670
Pheochromocytoma & Paraganglioma	187	161	685/27,114
Prostate Cancer	551	257	881/32,350
Rectal Cancer	177	81	923/33,955
Thyroid Cancer:	574	414	803/29,825

Table S2: Sample and gene counts for the 20 TCGA pan cancer cohorts used in the analysis. Note that for the number of mutation and CNV predictors, the same gene may be counted twice (i.e., both non-silent somatic mutation data and CNV data is available for the gene).

The TCGA gene expression and gene-level somatic alteration data was specifically drawn from the following data sets:

- TCGA gene expression data ($\mathbf{E_k}$)
  - **Source file**: HiSeqV2_PANCAN-2015-02-15.tgz
  - **Data type description (from UCSC Cancer Browser documentation)**: "TCGA pan-cancer gene expression by RNAseq. Gene expression measured using the IlluminaHiSeq technology. Data from all TCGA cohorts are combined to produce this dataset."
  - **Number of samples**: 9,755
- Gene-level non-silent somatic mutations ($\mathbf{M_k}$)
  - **Source file**: TCGA_PANCAN_mutation_xena_gene-2015-01-28.tgz
  - **Data type details (from UCSC Cancer Browser documentation)**: "TCGA pan-cancer somatic mutation data compiled from all cohorts mutation calls are available. Red (=1) indicates that a non-silent somatic mutation (nonsense, missense, frame-shif indels, splice site mutations, stop codon readthroughs) was identified in the protein coding region of a gene, or any mutation identified in a non-coding gene. White (=0) indicates that none of the above mutation calls were made in this gene for the specific sample. Somatic mutations calls (even on the same tumor DNA extract) are affected by many factors including library prep, sequencing process, read mapping method, reference genome used, genome annotation, calling algorithms, and ad-hoc pre/postprocessing such as black list genes, target selection regions, and black list samples. This dataset is the effort of the UCSC Xena team. Individual mutation data can be downloaded at TCGA DCC."
  - **Number of samples**: 6,901
- Gene-level CNV data ($\mathbf{C_k}$)
  - **Source file**: TCGA_PANCAN_gistic2-2015-02-06.tgz
  - **Data type details (from UCSC Cancer Browser documentation)**: "TCGA pan-cancer gene-level copy number variation (CNV) estimated using the GISTIC2 method. Copy number profile was measured experimentally using whole genome microarray at Broad TCGA genome characterization center. Subsequently, TCGA FIREHOSE pipeline applied GISTIC2 method to produce segmented CNV data, which was then mapped to genes to produce gene-level estimates. Gistic2 data from all TCGA cohorts are combined to produce this dataset. Reference to GISTIC2 method PMID:21527027." (Mermel *et al.*, 2011)
  - **Number of samples**: 10,843

1.3 Analysis pipeline

This section provides mathematical and implementation details for three analysis steps described in the Methods Section of the main manuscript. R code implementing this pipeline is available at http://www.dartmouth.edu/~hrfrost/MutPath/. It is assumed that the analysis is performed separately for each cancer type $k, k \in 1...t$.

- **Step 1**: For each of the m pathways defined in $\mathbf{A}$ (1), determine the extent to which expression of the pathway genes in each individual tumor varies from the mean expression for all n_k tumors of that cancer type. This computation is performed using the unsupervised, single-sample gene set testing method GSVA (Hänzelmann *et al.*, 2013). This analysis generates an $n_k \times m$ matrix $\mathbf{S_k}$ of single-subject pathway enrichment scores:

$$\mathbf{S_k} = \begin{bmatrix} s_{1,1} & \cdots & s_{1,m} \\ \vdots & \ddots & \vdots \\ s_{n_k,1} & \cdots & s_{n_k,m} \end{bmatrix} \quad (8)$$

  where $s_{i,j}$ holds the enrichment score computed by GSVA for pathway j using the gene expression data for tumor i from cancer type k held in row i of $\mathbf{E_k}$. Specifically, this matrix will contain normalized enrichment scores generated using the scoring procedure defined by Eq. 5 in Hanzelmann et al. (Hänzelmann *et al.*, 2013), which, under the null of no deviation from the mean pathway activity level for that cancer type, have a standard normal distribution, i.e., $s_{i,j}|H_0 \sim \mathcal{N}(0, 1)$.

If desired, the $\mathbf{S_k}$ matrix can be generated using alternative single sample gene set testing methods (e.g., ssGSEA (Barbie *et al.*, 2009)) or variations of the GSVA method (e.g., the GSVA statistic in Eq. 4 of Hanzelman et al. (Hänzelmann *et al.*, 2013) that can identify gene sets containing both up-regulated and down-regulated genes). Researchers interested in enabling such a change using the R implementation of our approach available at http://www.dartmouth.edu/∼hrfrost/MutPath/ can edit the GSVAUtils.R file. Importantly, the GSVA R package leveraged by our implementation also provides implementations of the ssGSEA (Barbie *et al.*, 2009), zscore (Lee *et al.*, 2008), and PLAGE (Tomfohr *et al.*, 2005) methods. To illustrate the impact of alternate single sample methods, tables S24 and S25 below show the top driver genes identified for the TCGA pancreatic cancer cohort using either GSVA or ssGSEA to generate single sample gene set scores. As demonstrated by these results, our approach is able to successfully identify known driver genes using either method (GSVA identified more known drivers in the top 20 in this case).

- **Step 2**: For each pathway $j, j = 1, ..., m$, fit both penalized and unpenalized multiple linear regression models using column j of the enrichment score matrix , $\mathbf{S_k}$, as the dependent variable and the $pm_k + pc_k$ predictors representing gene-level somatic mutation and CNV values:

$$E[\mathbf{S_k}[,\mathbf{j}]|\mathbf{M_k}, \mathbf{C_k}] = \beta_0 + \mathbf{M_k}\boldsymbol{\beta_m} + \mathbf{C_k}\boldsymbol{\beta_c} \tag{9}$$

where

  - β_0 captures the expected pathway enrichment score for this cancer type when none of the predictor genes contain non-silent somatic mutations or have copy number variation.
  - $\boldsymbol{\beta_m}$ is a length pm_k vector of regression coefficients for the indicators of gene-level non-silent somatic mutations, i.e., the effect of non-silent somatic mutations on pathway enrichment.
  - $\boldsymbol{\beta_c}$ is a length pc_k vector of regression coefficients for the CNV values, i.e., the effect of copy number variation on pathway enrichment.

For the reported analyzes, the penalized and unpenalized regression models were fit separately for both potential sets of the $pm_k + pc_k$ somatic alteration predictors. In other words, two pairs of penalized and unpenalized models were estimated for each pathway. Models were also estimated using randomized somatic alteration predictors, i.e., the subject labels for the predictors were randomly permuted. The first pair of models used as predictors the somatic alteration variables for all of the genes with a known cancer association, i.e., the b genes defined by $\mathbf{o}$ (3) (4). The second set of models used as predictors the somatic alteration variables for all available genes in the TCGA data. For the 20 analyzed TCGA cancer types, the number of somatic mutation and CNV predictors for both cases are listed in the third column of Table S2.

Because the number of predictors, $pm_k + pc_k$, will most likely be larger than the number of samples, n, the model in Eq (9) is initially fit using a LASSO (Tibshirani, 2011) penalty, which performs both variable selection and coefficient shrinkage. Because lasso-penalization tends to retain just one predictor from among a set of correlated predictors (Tibshirani, 2011), penalized estimation will tend to select a parsimonious group of gene-level somatic mutation and CNV variables with minimal overlap. Actual estimation for the reported results was performed using the *glmnet* R package (Friedman *et al.*, 2010). LASSO-penalized estimation is accomplished by maximizing the following objective function:

$$-\frac{log(L(\beta_0, \boldsymbol{\beta_m}, \boldsymbol{\beta_c}|\mathbf{M_k}, \mathbf{C_k})}{n} + \lambda(\sum_{i=1}^{pm_k} |\boldsymbol{\beta_m}[i]| + \sum_{i=1}^{pc_k} |\boldsymbol{\beta_c}[i]|) \tag{10}$$

Note that the intercept term, β_0, is not penalized in this objective function. The LASSO penalty parameter λ can be selected according to cross-validation or to achieve a specific number of non-zero coefficients. For the reported analyses, λ was set to the value that minimized mean squared error during 10-fold cross-validation with the cross-validation (CV) process repeated 5 times to reduce the variance associated with the random splitting of the data.

The CV results were also used to estimate the predicted coefficient of determination for the model (i.e., R^2_{pred}). Specifically, R^2_{pred} was estimated as the average proportion of null deviance explained by the lasso-penalized models on held-out data for each CV fold with the average value computed across all 50 total estimates, i.e., 5 replications of 10-fold CV. Note that the proportion of null deviance explained by the model is equivalent

and the coefficient of determination for multiple linear regression. Let the estimated R^2_{pred} values for all cancer types for a given gene set collection be held in a $t \times m$ matrix $\mathbf{R}$:

$$\mathbf{R} = \begin{bmatrix} r_{1,1} & \cdots & s_{1,m} \\ \vdots & \ddots & \vdots \\ s_{t,1} & \cdots & r_{t,m} \end{bmatrix} \tag{11}$$

where $r_{i,j}$ holds the R^2_{pred} estimated via cross-validation of the LASSO-penalized model for pathway j using the data the genomic data for cancer type i. For the reported analysis, 6 versions of the $\mathbf{R}$ matrix were generated:

– $\mathbf{R_{C2.CP,COSMIC}}$: holds the R^2_{pred} values for the MSigDB C2.CP pathway models using as predictors just the somatic alterations associated with COSMIC consensus cancer genes, i.e., the b genes defined by $\mathbf{o}$ (3) (4).

– $\mathbf{R_{C2.CP,COSMIC,random}}$: holds the R^2_{pred} values for the MSigDB C2.CP pathway models using as predictors just the somatic alterations associated with COSMIC consensus cancer genes with the association between subjects and predictor values randomly permuted.

– $\mathbf{R_{C2.CP,all}}$: holds the R^2_{pred} values for the MSigDB C2.CP pathway models using as predictors somatic alterations for all available TCGA genes.

– $\mathbf{R_{C6,COSMIC}}$: holds the R^2_{pred} values for the MSigDB C6 pathway models using as predictors just the somatic alterations associated with COSMIC consensus cancer genes, i.e., the b genes defined by $\mathbf{o}$ (3) (4).

– $\mathbf{R_{C6,COSMIC,random}}$: holds the R^2_{pred} values for the MSigDB C6 pathway models using as predictors just the somatic alterations associated with COSMIC consensus cancer genes with the association between subjects and predictor values randomly permuted.

– $\mathbf{R_{C6,all}}$: holds the R^2_{pred} values for the MSigDB C6 pathway models using as predictors somatic alterations for all available TCGA genes.

Following penalized estimation, an unpenalized multiple linear regression is performed for model (9) using as dependent variables just the subset of the $pm_k + pc_k$ somatic alteration predictors that have non-zero coefficient estimates at the optimal λ from the penalized regression. Let the p-values associated with the t-statistics for the estimated coefficients in the unpenalized linear models for cancer type k be held in a $m \times (pm_k + pc_k)$ matrix $\mathbf{Pval_k}$:

$$\mathbf{Pval_k} = \begin{bmatrix} pval_{1,1} & \cdots & pval_{1,pm_k+pc_k} \\ \vdots & \ddots & \vdots \\ pval_{m,1} & \cdots & pval_{m,pm_k+pc_k} \end{bmatrix} \tag{12}$$

where $pval_{i,j}$ holds the p-value associated with the estimated coefficient for somatic alteration predictor j in the unpenalized regression model for pathway i and cancer type k. Note that the elements of this matrix associated with predictors that had 0 coefficients in the LASSO-penalized models will be undefined. For the reported results, 4 different versions of the $\mathbf{Pval_k}$ matrix were generated (one for each of the 4 possible combinations of MSigDB collection and somatic alteration predictor sets).

- **Step 3**: Use the fitted regression models to address one of the three primary aims. The Methods and Results Sections in the main manuscript contain details on the motivation, expectations and results for the evaluation procedures. Mathematical and implementation details for the evaluation results shown in the figures and tables of the main manuscript and SI are detailed in the next section.

1.4 Analysis of breast cancer subtypes

To determine if the pathway models fit for the high-level TCGA cancer types could be used to characterize the features of cancer subtypes, we explored the differences in predicted pathway activity and somatic mutation predictors for the TCGA BRCA subjects assigned to either the basal (n=124) or luminal (n=667) PANCAN cluster-of-cluster assignments Hoadley *et al.* (2014). To identify pathways whose activity differed between basal and luminal subtypes, the following steps were performed:

- For each of the pathways in either the C2.CP or C6 collection, the unpenalized regression model fit according to the approach outlined above was used to generate a predicated pathway dysregulation score for each breast cancer subject using that subject's somatic alteration values (i.e., the non-silent somatic mutation indicators and CNV estimates for all model predictors with non-zero effect sizes).

- For each pathway, a two-sample t-test was performed comparing the predicated pathway scores for luminal tumors with the predicated pathway scores for basal tumors.
- The pathways were then ranked according to the estimated t-statistics with large positive statistics representing pathways with greater activity in luminal tumors and with large negative statistics representing greater activity in basal tumors.

To identify somatic alteration predictors whose importance in driving pathway activity differs between basal and luminal subtypes, the following steps were performed:

- For each somatic mutation or CNV predictor included in at least one pathway model, a subject-level score was computed by taking the sum across all pathway models of the product of the following values:
  - The absolute value of subject's measurement for that predictor.
  - The -log of the p-value for that predictor in the unpenalized regression model.
  - The predicted R^2 value for the pathway model.
- The average of the predictor scores was computed among the luminal subjects and separately among the basal subjects.
- The predictors were then ranked according to the difference between the mean luminal and mean basal scores with large positive mean differences corresponding to somatic alterations that are more important in driving pathway activity among luminal tumors and with large negative mean differences corresponding to somatic alterations that are more important in driving pathway activity among basal tumors.

The results of these comparative pathway and predictor analyses are contained in Tables S18-S23 below.

1.5 Generation of tables and figures

The tables and figures included in the main manuscript and SI were generated as detailed below. Associated R logic is available at http://www.dartmouth.edu/∼hrfrost/MutPath/.

- **Heatmap of R^2_{pred} values:** For Figure 2 in the main manuscript, the heatmap was generated using the R^2_{pred} values in the matrix $\mathbf{R_{C2.CP,COSMIC}}$ with both the columns (i.e., pathways) and rows (i.e., cancer types) ordered according to the results of agglomerative hierarchical clustering using complete link agglomeration and euclidean distance. The heatmap shown in Figure S1 was generating using a similar procedure for the R^2_{pred} values in the matrix $\mathbf{R_{C6,COSMIC}}$.
- **Box plots of R^2_{pred} values:** For Figure 3 in the main manuscript, the box plot was generated using the R^2_{pred} values in the following matrices: $\mathbf{R_{C2.CP,COSMIC}}$, $\mathbf{R_{C2.CP,COSMIC,random}}$ and $\mathbf{R_{C2.CP,all}}$. For Figure S2, the box plot was generated using the R^2_{pred} values in $\mathbf{R_{C6,COSMIC}}$, $\mathbf{R_{C6,COSMIC,random}}$ and $\mathbf{R_{C6,all}}$
- **Rank correlation between R^2_{pred} values for different predictor sets:** For Figure 4 in the main manuscript, the correlation value for TCGA cancer type k was computed as the Spearman rank correlation between row k of matrix $\mathbf{R_{C2.CP,COSMIC}}$ and row k of matrix $\mathbf{R_{C2.CP,all}}$. For Figure S3, the correlation value for TCGA cancer type k was computed as the Spearman rank correlation between row k of matrix $\mathbf{R_{C6,COSMIC}}$ and row k of matrix $\mathbf{R_{C6,all}}$.
- **Ranking of pathway models by R^2_{pred}:** Table 1 in the main manuscript shows the top 10 MSigDB C2.CP pathway models according to the R^2_{pred} values estimated for the TCGA lung adenocarcinoma data. The rank ordering is displayed for models estimated using both potential sets of predictors. Specifically, the left side is ordered according to the R^2_{pred} values in the row of $\mathbf{R_{C2.CP,COSMIC}}$ corresponding to lung adenocarcinoma and the right side is ordered according to the R^2_{pred} values in the row of $\mathbf{R_{C2.CP,all}}$ corresponding to lung adenocarcinoma. The # columns specify the rank of that pathway according to the R^2_{pred} values in $\mathbf{R_{C2.CP,COSMIC}}$. Similar tables displaying the top 20 pathways from the MSigDB C2.CP and C6 collections are included in the SI for the fiveTCGA cohorts with the largest number of samples that have expression, somatic mutation and CNV data: breast cancer (Tables S3 and S4), head and neck cancer (Tables S6 and S7), lower grade glioma (Tables S9 and S10), lung adenocarcinoma (Tables S12 and S13) and thyroid cancer (Tables S15 and S16).

- **Mean R^2_{pred} values:** Figures S4 and S5 illustrate the association between cohort sample size and mean R^2_{pred} values for each TCGA cancer type. For Figure S4, the mean R^2_{pred} values are computed for each row of matrix $\mathbf{R_{C2.CP,COSMIC}}$. For Figure S5, the mean R^2_{pred} values are computed for each row of matrix $\mathbf{R_{C6,COSMIC}}$

- **Ranking of somatic alteration predictors:** The individual gene-level somatic mutation and CNV predictors where rank ordered for each TCGA cohort according to a weight w computed the sum average across all pathway models for a specific MSigDB collection of the product of the -log(p-value) from the unpenalized regression model and the R^2_{pred} estimate for the penalized model. If the index of the predictor among all $pm_k + pc_k$ predictors is e, then w is computed for cancer type k according to the following formula:

$$w = \frac{1}{m} \sum_{i=1}^{m} -log(\mathbf{Pval_k}[i, e])\mathbf{R}[k, i] \tag{13}$$

  All predictor weights can be held in a $t \times (pm_k + pc_k)$ matrix W:

$$\mathbf{W} = \begin{bmatrix} w_{1,1} & \cdots & w_{1,pm_k+pc_k} \\ \vdots & \ddots & \vdots \\ w_{t,1} & \cdots & w_{t,pm_k+pc_k} \end{bmatrix} \tag{14}$$

  where $wl_{i,j}$ holds the weight for predictor j and cancer type i. For the reported results, 4 different versions of W were computed:

  – $\mathbf{W_{C2.CP,COSMIC}}$: The weights are computed across the C2.CP pathways estimated using just the COSMIC cancer consensus gene predictors.
  – $\mathbf{W_{C2.CP,all}}$: The weights are computed across the C2.CP pathways estimated using all available TCGA genes as predictors.
  – $\mathbf{W_{C6,COSMIC}}$: The weights are computed across the C6 pathways estimated using just the COSMIC cancer consensus gene predictors.
  – $\mathbf{W_{C6,all}}$: The weights are computed across the C6 pathways estimated using all available TCGA genes as predictors.

  Table 2 in the main manuscript shows the top 10 somatic alteration predictors ranked according to the w values in the lung adenocarcinoma row of the $\mathbf{W_{C2.CP,COSMIC}}$, $\mathbf{W_{C6,COSMIC}}$, $\mathbf{W_{C2.CP,all}}$ and $\mathbf{W_{C6,all}}$ matrices. Similar tables showing the top 20 predictors are included in the SI for the five TCGA cohorts with the largest number of samples that have expression, somatic mutation and CNV data: breast cancer (Table S5), head and neck cancer (Table S8), lower grade glioma (Table S11), lung adenocarcinoma (Table S14) and thyroid cancer (Table S17).

- **Rank correlation between somatic alteration predictors for different MSigDB collections:** For Figure 6 in the main manuscript, the correlation value for TCGA cancer type k was computed as the Spearman rank correlation between row k of matrix $\mathbf{W_{C2.CP,COSMIC}}$ and row k of matrix $\mathbf{W_{C6,COSMIC}}$. For Figure S6, the correlation value for TCGA cancer type k was computed as the Spearman rank correlation between row k of matrix $\mathbf{W_{C2.CP,all}}$ and row k of matrix $\mathbf{W_{C6,all}}$,

- **Enrichment of known driver genes among top ranked predictors:** Figure 5 and S7 illustrate the enrichment of known driver genes for each cancer type among the predictors ranked according to the weights in $\mathbf{W}$. Specifically, for each cancer type k a Wilcoxon rank sum test was performed between the elements of row k of matrix $\mathbf{W}_{C2.CP,all}$ (or $\mathbf{W}_{C6,all}$) associated with genes that have non-zero values in row k of matrix $\mathbf{D}$ (i.e., genes with a known association with cancer type k) and the elements of row k of matrix $\mathbf{W}_{C2.CP,all}$ (or $\mathbf{W}_{C6,all}$) associated with genes that have zero values in row k of matrix $\mathbf{D}$ (i.e., genes with no known association with cancer type k). False discovery rate (FDR) q-values were computed on the Wilcoxon test p-values using the Benjamini and Hochberg (BH) (Benjamini and Hochberg, 1995) method for a family of hypotheses that includes all t cancer types. In Figure 5, the q-values for each TCGA cohort, as computed using the C2.CP versions of the $\mathbf{W}$ matrix, are plotted relative to cohort sample size. Figure S7 illustrates the q-values computed using the C6 versions of the $\mathbf{W}$ matrix.

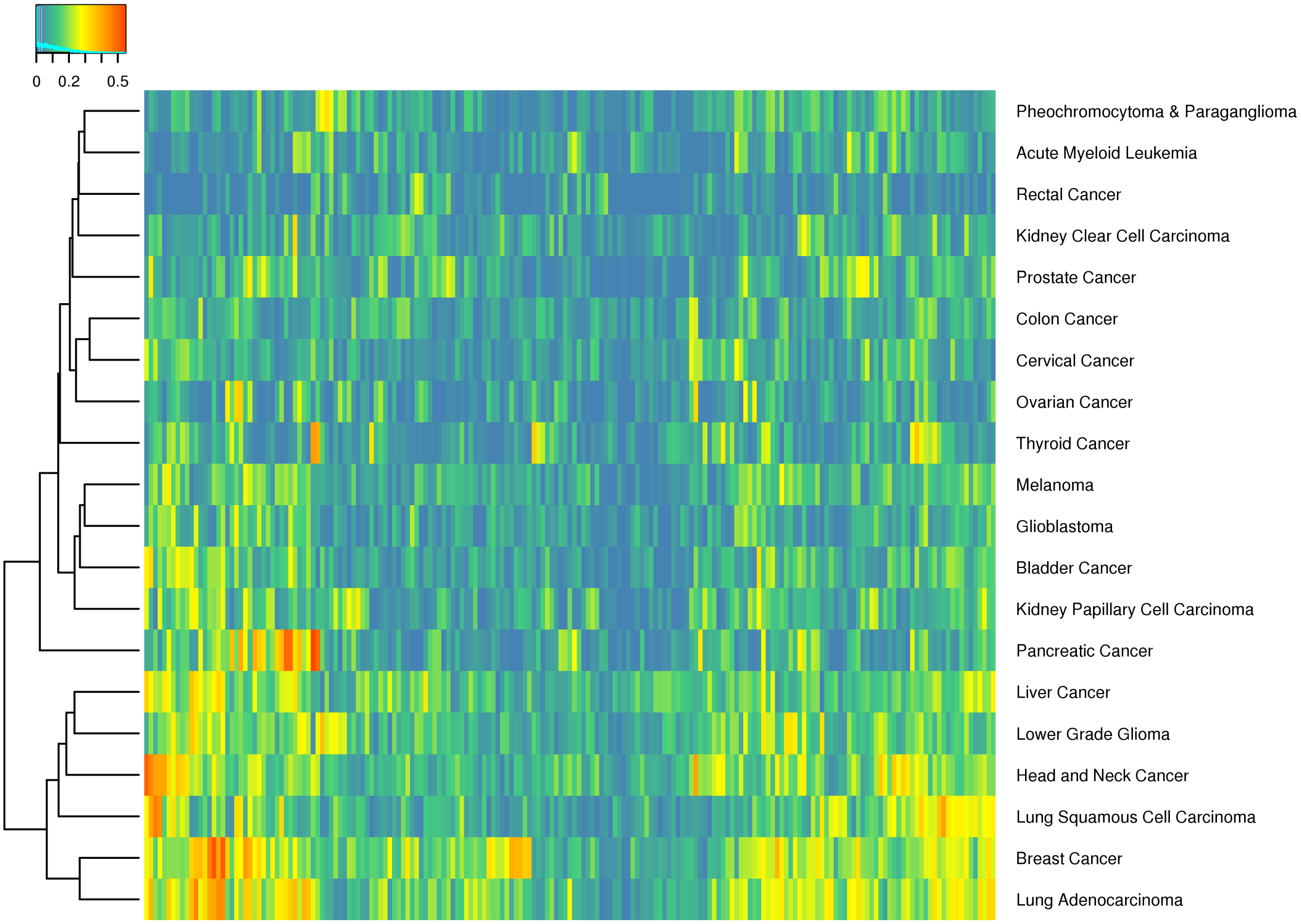

Figure S1: Illustration of results from the analysis of TCGA pan cancer data using the MSigDB oncogenic signatures (C6) collection. Rows correspond to the TCGA cohorts and columns to MSigDB gene sets. Each cell represents a pathway and cancer type-specific regression model colored according to the R^2_{pred} value. Both the columns and rows are ordered according to the output from hierarchical agglomerative clustering using complete link agglomeration and euclidean distance.

2 Supplemental Results

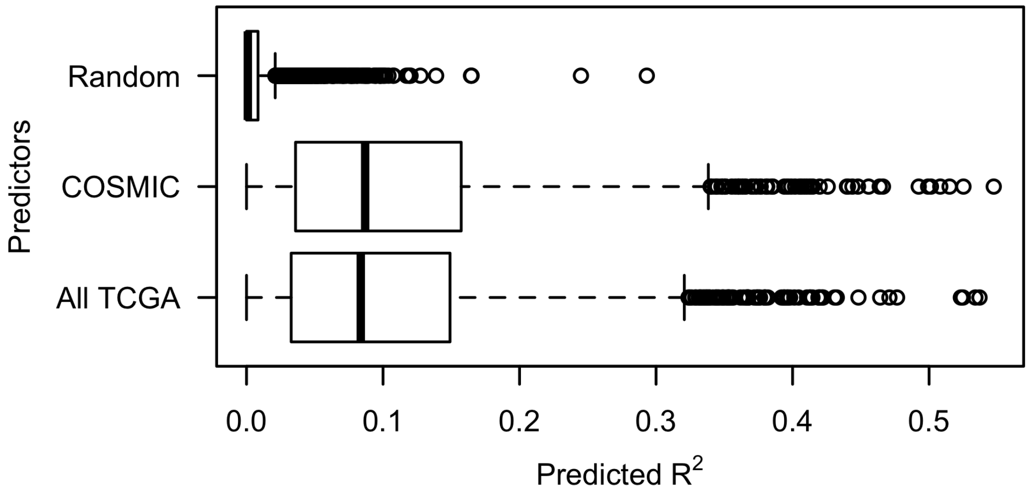

Figure S2: Box plot illustrating the distribution of R^2_{pred} values from the analysis of TCGA pan cancer data using the MSigDB oncogenic signatures (C6) collection. Values are shown for three different analyses: *COSMIC*: estimation of pathway regression models using somatic alteration data for COSMIC consensus cancer genes, *All TCGA*: estimation of models using somatic alteration data for all genes included in the TCGA datasets, and *Random*: estimation of models using randomized somatic alteration data for COSMIC consensus cancer genes.

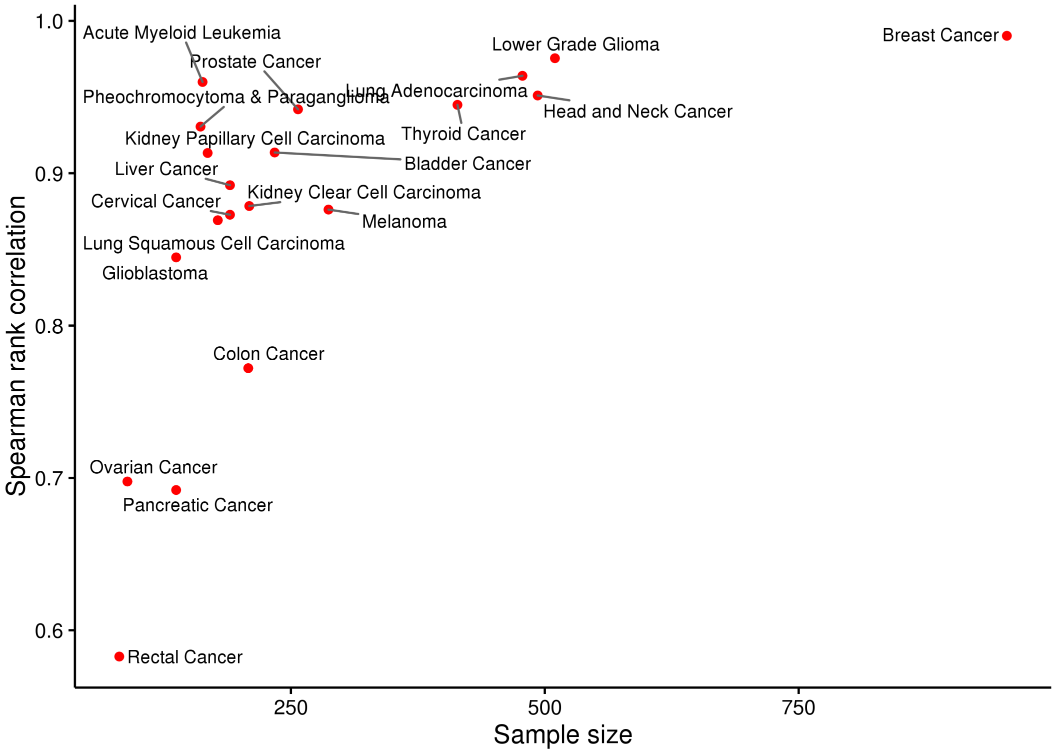

Figure S3: Spearman rank correlation between R^2_{pred} values for C6 pathway models estimated using just COSMIC consensus cancer genes and the R^2_{pred} values for C2.CP pathway models estimated using all TCGA genes. The rank correlation values are computed separately for each TCGA cohort and are plotted relative to cohort sample size.

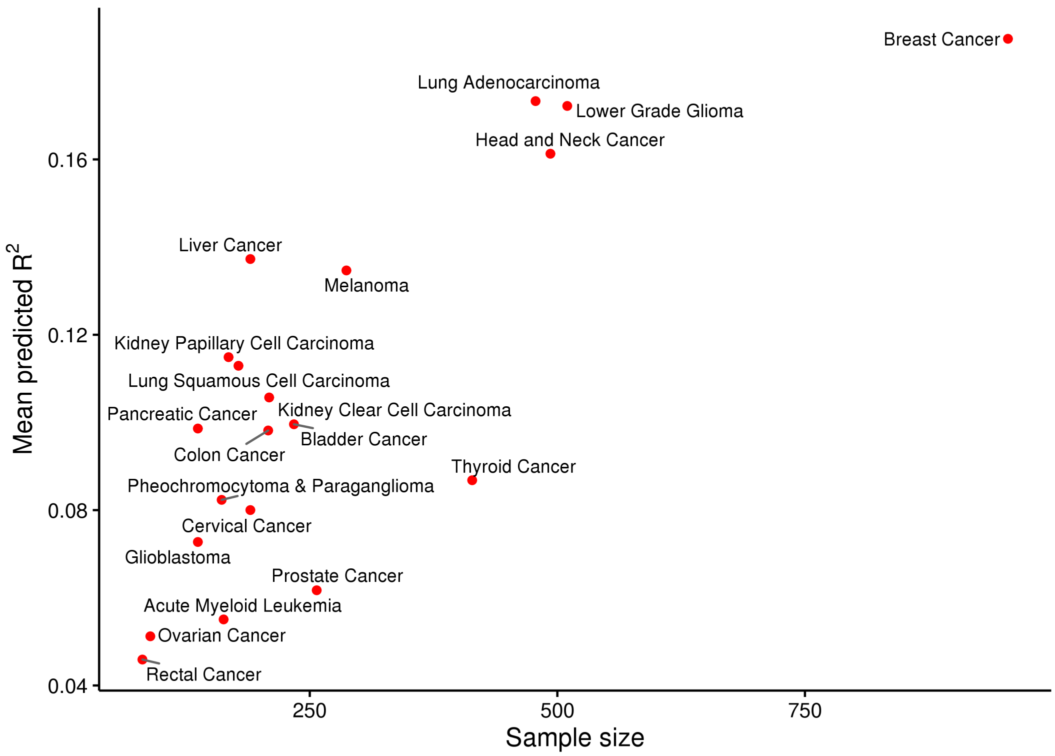

Figure S4: Mean R^2_{pred} values for C2.CP pathway models estimated for each TCGA cohort using just COSMIC consensus cancer genes. The mean R^2_{pred} values are plotted relative to cohort sample size.

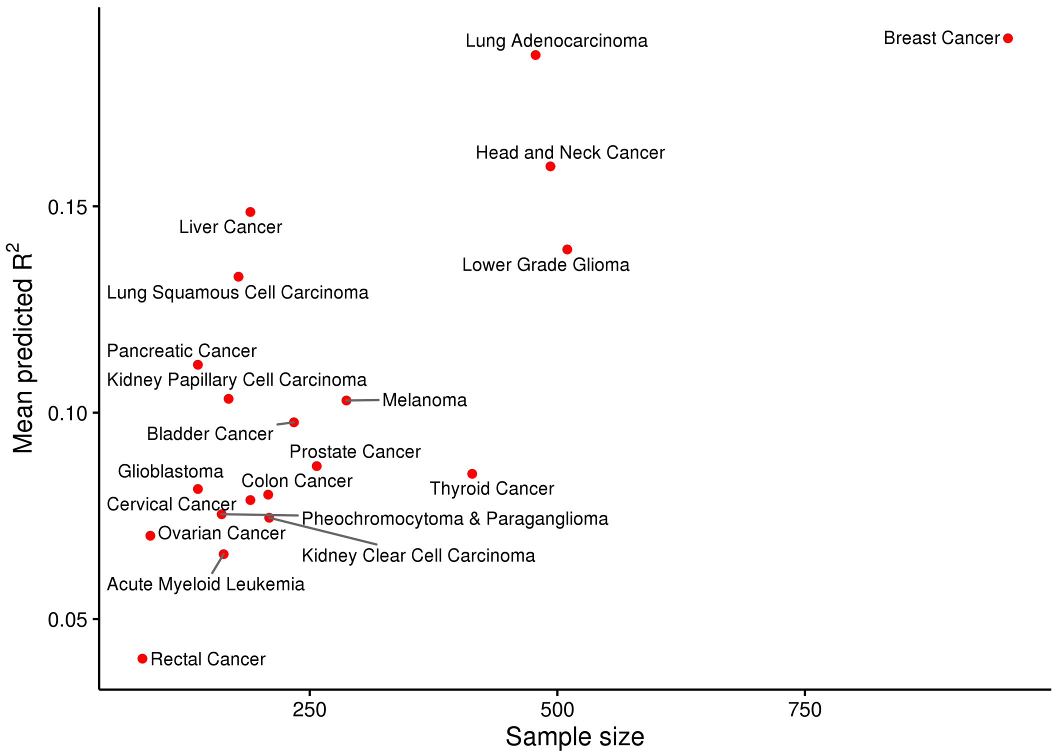

Figure S5: Mean R^2_{pred} values for C6 pathway models estimated for each TCGA cohort using just COSMIC consensus cancer genes. The mean R^2_{pred} values are plotted relative to cohort sample size.

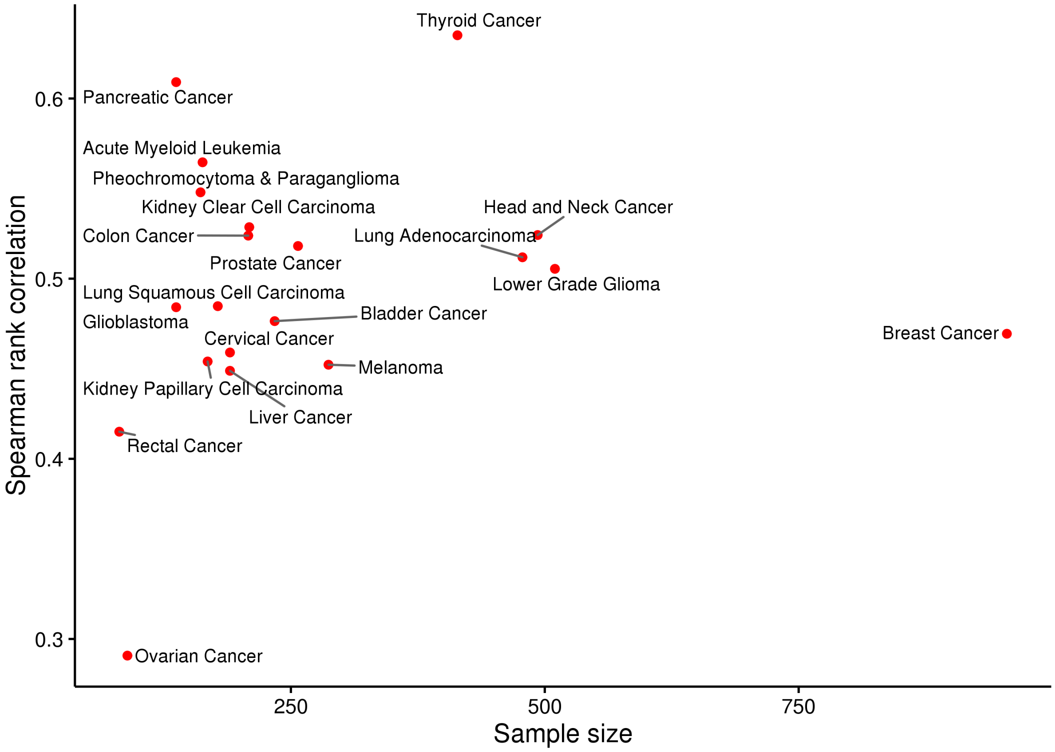

Figure S6: Spearman rank correlation between the weights for somatic alteration predictors for all available TCGA genes computed for the MSigDB C2.CP and C6 collections. The rank correlation values are computed separately for each TCGA cohort and are plotted relative to cohort sample size.

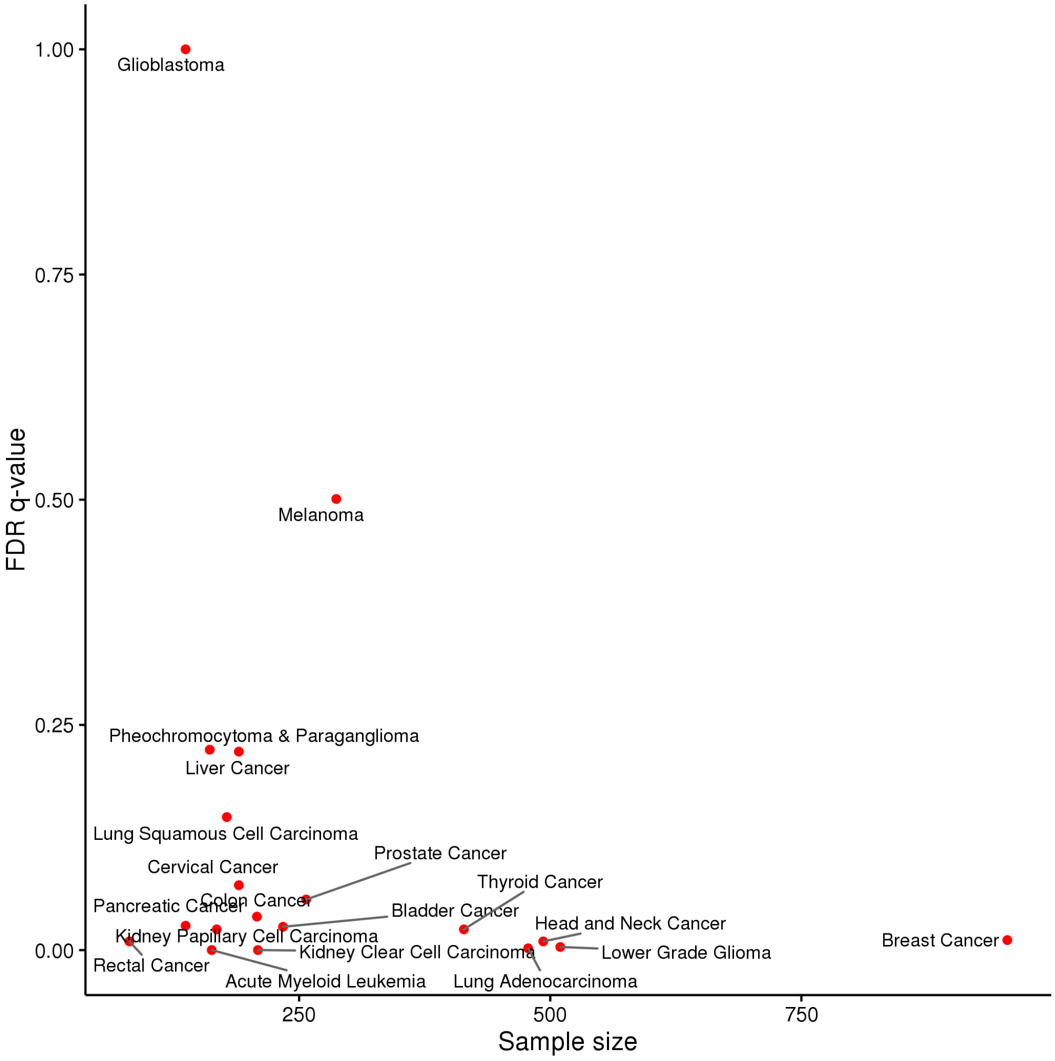

Figure S7: The false discovery rate (FDR) q-values (as computed using the Benjamini and Hochberg (BH) (Benjamini and Hochberg, 1995) method) for the Wilcox rank sum tests of enrichment of known driver genes among the ranks of all predictors for the MSigDB C6 models using all TCGA genes. The q-value for each cancer type cohort is plotted relative to the cohort sample size.

Table S3: Top MSigDB C2.CP pathway models for Breast Cancer

Consensus cancer genes			All TCGA genes		
#	Pathway	R^2_{pred}	#	Pathway	R^2_{pred}
1	BIOCARTA_RANMS_PATHWAY	0.481	1	BIOCARTA_RANMS_PATHWAY	0.48
2	SA_REG_CASCADE_OF_CYCLIN_EXPR	0.48	2	SA_REG_CASCADE_OF_CYCLIN_EXPR	0.465
3	REACTOME_GO_AND_EARLY_G1	0.457	3	REACTOME_GO_AND_EARLY_G1	0.463
4	REACTOME_UNWINDING_OF_DNA	0.456	4	REACTOME_UNWINDING_OF_DNA	0.446
5	REACTOME_CDC6_ASSOCIATION_WITH_THE_ORC_ORIGIN_COMP...	0.45	8	REACTOME_MITOTIC_M_M_G1_PHASES	0.444
6	REACTOME_ASSOCIATION_OF_LICENSING_FACTORS_WITH_THE...	0.443	10	REACTOME_DNA_REPLICATION	0.44
7	PID_MYC_ACTIV_PATHWAY	0.441	5	REACTOME_CDC6_ASSOCIATION_WITH_THE_ORC_ORIGIN_COMP...	0.438
8	REACTOME_MITOTIC_M_M_G1_PHASES	0.44	6	REACTOME_ASSOCIATION_OF_LICENSING_FACTORS_WITH_THE...	0.438
9	REACTOME_KINESINS	0.439	9	REACTOME_KINESINS	0.432
10	REACTOME_DNA_REPLICATION	0.437	30	BIOCARTA_SRCRPTP_PATHWAY	0.43
11	REACTOME_CELL_CYCLE	0.428	12	REACTOME_PROCESSING_OF_CAPPED_INTRONLESS_PRE_MRNA	0.429
12	REACTOME_PROCESSING_OF_CAPPED_INTRONLESS_PRE_MRNA	0.427	14	REACTOME_CLEAVAGE_OF_GROWING_TRANSCRIPT_IN_THE_TER...	0.427
13	KEGG_CELL_CYCLE	0.427	7	PID_MYC_ACTIV_PATHWAY	0.427
14	REACTOME_CLEAVAGE_OF_GROWING_TRANSCRIPT_IN_THE_TER...	0.426	15	PID_FOXM1_PATHWAY	0.427
15	PID_FOXM1_PATHWAY	0.424	18	REACTOME_G1_S_SPECIFIC_TRANSCRIPTION	0.426
16	REACTOME_RAF_MAP_KINASE_CASCADE	0.422	31	REACTOME_E2F_ENABLED_INHIBITION_OF_PRE_REPLICATION...	0.424
17	REACTOME_SOS_MEDIATED_SIGNALLING	0.42	11	REACTOME_CELL_CYCLE	0.423
18	REACTOME_G1_S_SPECIFIC_TRANSCRIPTION	0.416	13	KEGG_CELL_CYCLE	0.413
19	REACTOME_CELL_CYCLE_MITOTIC	0.416	21	PID_AURORA_B_PATHWAY	0.412
20	REACTOME_SYNTHESIS_OF_GLYCOSYLPHOSPHATIDYLINOSITOL...	0.411	24	BIOCARTA_MCM_PATHWAY	0.412

Table S4: Top MSigDB C6 pathway models for Breast Cancer

Consensus cancer genes			All TCGA genes		
#	Pathway	R^2_{pred}	#	Pathway	R^2_{pred}
1	HOXA9_DN.V1_DN	0.525	2	RPS14_DN.V1_DN	0.537
2	RPS14_DN.V1_DN	0.515	1	HOXA9_DN.V1_DN	0.523
3	CSR_LATE_UP.V1_UP	0.466	3	CSR_LATE_UP.V1_UP	0.477
4	LEF1_UP.V1_DN	0.426	4	LEF1_UP.V1_DN	0.422
5	E2F1_UP.V1_UP	0.41	5	E2F1_UP.V1_UP	0.419
6	RAF_UP.V1_DN	0.397	7	PRC2_SUZ12_UP.V1_DN	0.401
7	PRC2_SUZ12_UP.V1_DN	0.385	8	MTOR_UP.N4.V1_UP	0.394
8	MTOR_UP.N4.V1_UP	0.385	6	RAF_UP.V1_DN	0.392
9	CSR_EARLY_UP.V1_UP	0.381	10	CSR_EARLY_UP.V1_DN	0.38
10	CSR_EARLY_UP.V1_DN	0.381	11	MTOR_UP.V1_UP	0.379
11	MTOR_UP.V1_UP	0.377	9	CSR_EARLY_UP.V1_UP	0.368
12	PRC2_EZH2_UP.V1_UP	0.364	12	PRC2_EZH2_UP.V1_UP	0.368
13	EGFR_UP.V1_DN	0.358	15	RELA_DN.V1_UP	0.362
14	YAP1_DN	0.348	13	EGFR_UP.V1_DN	0.349
15	RELA_DN.V1_UP	0.344	17	IL15_UP.V1_DN	0.344
16	RB_P107_DN.V1_UP	0.34	14	YAP1_DN	0.338
17	IL15_UP.V1_DN	0.334	19	IL2_UP.V1_DN	0.329
18	GCNP_SHH_UP_LATE.V1_UP	0.324	18	GCNP_SHH_UP_LATE.V1_UP	0.319
19	IL2_UP.V1_DN	0.322	16	RB_P107_DN.V1_UP	0.318
20	PDGF_UP.V1_DN	0.321	24	MTOR_UP.N4.V1_DN	0.313

Table S5: Top predictors for Breast Cancer

Consensus cancer genes						All TCGA genes					
C2.CP			C6			C2.CP			C6		
#	Predictor	W	#	Predictor	W	#	Predictor	W	#	Predictor	W
1	**TP53**	0.971	2	**CDH1**	1.1	1	**TP53**	0.856	2	**CDH1**	1.18
2	**CDH1**	0.741	1	**TP53**	1.08	2	**CDH1**	0.765	1	**TP53**	0.989
3	**GATA3**	0.389	3	**GATA3**	0.434	3	**PIK3CA**	0.3	3	**PIK3CA**	0.372
4	**CCND1 (CNV)**	0.387	5	**RB1 (CNV)**	0.429	4	**GATA3**	0.27	4	**GATA3**	0.314
5	**RB1 (CNV)**	0.355	4	**CCND1 (CNV)**	0.388	5	*TTN	0.229	5	*TTN	0.191
6	**ERBB2 (CNV)**	0.306	6	**ERBB2 (CNV)**	0.383	6	*RN7SKP201 (CNV)	0.09	15	*APOB	0.141
7	MYC (CNV)	0.299	7	MYC (CNV)	0.351	7	*WWOX (CNV)	0.0808	8	*DEFB132 (CNV)	0.123
8	**EP300 (CNV)**	0.27	10	**PIK3CA**	0.348	8	*DEFB132 (CNV)	0.0794	16	*RN7SKP226 (CNV)	0.0961
9	NUMA1 (CNV)	0.269	11	PCM1 (CNV)	0.215	9	*SHANK2 (CNV)	0.0787	6	*RN7SKP201 (CNV)	0.0908
10	**PIK3CA**	0.266	14	KAT6A (CNV)	0.215	10	*TSPAN14 (CNV)	0.0755	9	*SHANK2 (CNV)	0.0908
11	PCM1 (CNV)	0.225	8	**EP300 (CNV)**	0.19	11	*ZNF703 (CNV)	0.0751	40	*MIR873 (CNV)	0.0882
12	ERCC5 (CNV)	0.206	24	SPECC1 (CNV)	0.19	12	*MIR4300 (CNV)	0.0742	42	*TACC1 (CNV)	0.0855
13	MAML2 (CNV)	0.203	15	FGFR1 (CNV)	0.187	13	*CCDC171 (CNV)	0.0703	23	*MLL3	0.0843
14	KAT6A (CNV)	0.197	9	NUMA1 (CNV)	0.184	14	**RB1 (CNV)**	0.0668	7	*WWOX (CNV)	0.0737
15	FGFR1 (CNV)	0.191	57	MYOD1 (CNV)	0.181	15	*APOB	0.0661	21	*ACACA (CNV)	0.0734
16	PRKAR1A (CNV)	0.188	38	FUS (CNV)	0.17	16	*RN7SKP226 (CNV)	0.0603	48	*ZNF217 (CNV)	0.0712
17	IRF4 (CNV)	0.181	21	CDKN2A (CNV)	0.169	17	*PVT1 (CNV)	0.0591	26	*NOL11 (CNV)	0.0696
18	FLCN (CNV)	0.175	13	MAML2 (CNV)	0.167	18	**MAP3K1**	0.0578	14	**RB1 (CNV)**	0.0661
19	CLTC (CNV)	0.174	51	CLP1 (CNV)	0.163	19	*RNA5SP406 (CNV)	0.0573	19	*RNA5SP406 (CNV)	0.0655
20	CYLD (CNV)	0.173	27	EGFR (CNV)	0.154	20	*PPP6R3 (CNV)	0.0559	54	*NEUROD2 (CNV)	0.0646

Table S6: Top MSigDB C2.CP pathway models for Head and Neck Cancer

Consensus cancer genes			All TCGA genes		
#	Pathway	R^2_{pred}	#	Pathway	R^2_{pred}
1	REACTOME_THE_NLRP3_INFLAMMASOME	0.513	1	REACTOME_THE_NLRP3_INFLAMMASOME	0.496
2	REACTOME_INFLAMMASOMES	0.498	2	REACTOME_INFLAMMASOMES	0.467
3	BIOCARTA_TCAPOPTOSIS_PATHWAY	0.436	3	BIOCARTA_TCAPOPTOSIS_PATHWAY	0.441
4	BIOCARTA_NO2IL12_PATHWAY	0.433	16	SA_FAS_SIGNALING	0.433
5	PID_IL12_2PATHWAY	0.428	4	BIOCARTA_NO2IL12_PATHWAY	0.43
6	REACTOME_BASIGIN_INTERACTIONS	0.426	5	PID_IL12_2PATHWAY	0.429
7	REACTOME_INNATE_IMMUNE_SYSTEM	0.425	6	REACTOME_BASIGIN_INTERACTIONS	0.428
8	BIOCARTA_CTL_PATHWAY	0.421	9	REACTOME_IMMUNE_SYSTEM	0.424
9	REACTOME_IMMUNE_SYSTEM	0.42	10	KEGG_ALLOGRAFT_REJECTION	0.422
10	KEGG_ALLOGRAFT_REJECTION	0.42	12	REACTOME_PD1_SIGNALING	0.42
11	BIOCARTA_IL12_PATHWAY	0.418	8	BIOCARTA_CTL_PATHWAY	0.42
12	REACTOME_PD1_SIGNALING	0.417	11	BIOCARTA_IL12_PATHWAY	0.42
13	BIOCARTA_TH1TH2_PATHWAY	0.412	13	BIOCARTA_TH1TH2_PATHWAY	0.417
14	ST_IL_13_PATHWAY	0.41	17	REACTOME_ADAPTIVE_IMMUNE_SYSTEM	0.413
15	KEGG_NATURAL_KILLER_CELL_MEDIATED_CYTOTOXICITY	0.41	24	KEGG_GRAFT_VERSUS_HOST_DISEASE	0.411
16	SA_FAS_SIGNALING	0.409	19	PID_CD8_TCR_PATHWAY	0.404
17	REACTOME_ADAPTIVE_IMMUNE_SYSTEM	0.402	28	PID_TCR_PATHWAY	0.403
18	REACTOME_IMMUNOREGULATORY_INTERACTIONS_BETWEEN_A_L...	0.4	33	ST_INTERFERON_GAMMA_PATHWAY	0.399
19	PID_CD8_TCR_PATHWAY	0.397	7	REACTOME_INNATE_IMMUNE_SYSTEM	0.399
20	REACTOME_ANTIGEN_PRESENTATION_FOLDING_ASSEMBLY_AND...	0.393	18	REACTOME_IMMUNOREGULATORY_INTERACTIONS_BETWEEN_A_L...	0.398

Table S7: Top MSigDB C6 pathway models for Head and Neck Cancer

Consensus cancer genes			All TCGA genes		
#	Pathway	R^2_{pred}	#	Pathway	R^2_{pred}
1	E2F3_UP.V1_UP	0.508	1	E2F3_UP.V1_UP	0.464
2	PTEN_DN.V2_UP	0.448	2	PTEN_DN.V2_UP	0.422
3	RB_DN.V1_DN	0.414	5	HOXA9_DN.V1_UP	0.42
4	KRAS.DF.V1_UP	0.413	4	KRAS.DF.V1_UP	0.42
5	HOXA9_DN.V1_UP	0.409	6	NFE2L2.V2	0.394
6	NFE2L2.V2	0.405	3	RB_DN.V1_DN	0.375
7	BMI1_DN_MEL18_DN.V1_UP	0.36	9	STK33_NOMO_DN	0.352
8	TGFB_UP.V1_UP	0.35	7	BMI1_DN_MEL18_DN.V1_UP	0.335
9	STK33_NOMO_DN	0.35	15	CSR_EARLY_UP.V1_UP	0.333
10	PRC2_EZH2_UP.V1_DN	0.345	12	RPS14_DN.V1_UP	0.332
11	BMI1_DN.V1_UP	0.338	11	BMI1_DN.V1_UP	0.329
12	RPS14_DN.V1_UP	0.326	13	MEL18_DN.V1_UP	0.32
13	MEL18_DN.V1_UP	0.311	10	PRC2_EZH2_UP.V1_DN	0.317
14	SNF5_DN.V1_DN	0.306	8	TGFB_UP.V1_UP	0.315
15	CSR_EARLY_UP.V1_UP	0.298	17	GLI1_UP.V1_DN	0.292
16	HOXA9_DN.V1_DN	0.297	16	HOXA9_DN.V1_DN	0.291
17	GLI1_UP.V1_DN	0.294	23	LTE2_UP.V1_DN	0.283
18	HINATA_NFKB_MATRIX	0.288	29	SNF5_DN.V1_UP	0.279
19	ESC_V6.5_UP_EARLY.V1_DN	0.282	21	IL15_UP.V1_DN	0.278
20	TBK1.DF_UP	0.267	18	HINATA_NFKB_MATRIX	0.272

Table S8: Top predictors for Head and Neck Cancer

Consensus cancer genes						All TCGA genes					
C2.CP			C6			C2.CP			C6		
#	Predictor	W	#	Predictor	W	#	Predictor	W	#	Predictor	W
1	CDKN2A (CNV)	0.56	2	NSD1	0.6	1	TP53	0.342	1	TP53	0.413
2	NSD1	0.558	3	TP53	0.457	2	CASP8	0.29	2	CASP8	0.382
3	TP53	0.391	4	ASXL1 (CNV)	0.437	3	NSD1	0.258	4	*TTN	0.27
4	ASXL1 (CNV)	0.347	5	CASP8	0.425	4	*TTN	0.227	3	NSD1	0.262
5	CASP8	0.33	1	CDKN2A (CNV)	0.389	5	FBXW7	0.177	12	HRAS	0.184
6	FBXW7	0.266	9	CCND1 (CNV)	0.337	6	PIK3CA	0.144	13	**FAT1**	0.131
7	PIK3CA	0.231	22	HRAS	0.256	7	**NOTCH1**	0.116	8	CDKN2A	0.131
8	IKZF1 (CNV)	0.223	10	**NOTCH1**	0.233	8	CDKN2A	0.0945	16	**NFE2L2**	0.119
9	CCND1 (CNV)	0.212	6	FBXW7	0.22	9	*IL18BP (CNV)	0.0907	5	FBXW7	0.118
10	**NOTCH1**	0.203	21	PCM1 (CNV)	0.22	10	*C9orf53 (CNV)	0.0835	6	PIK3CA	0.109
11	NUMA1 (CNV)	0.202	8	IKZF1 (CNV)	0.217	11	*TP63 (CNV)	0.0826	53	LRP1B	0.106
12	ERBB2 (CNV)	0.195	13	RPL10 (CNV)	0.211	12	HRAS	0.0812	27	*PKHD1L1	0.104
13	RPL10 (CNV)	0.179	25	**FAT1**	0.196	13	**FAT1**	0.0809	11	*TP63 (CNV)	0.101
14	EGFR (CNV)	0.178	7	PIK3CA	0.192	14	*MACF1	0.0793	7	**NOTCH1**	0.0968
15	MYC (CNV)	0.174	12	ERBB2 (CNV)	0.191	15	*USH2A	0.072	122	*UNC5D (CNV)	0.0897
16	CBLB (CNV)	0.163	15	MYC (CNV)	0.19	16	**NFE2L2**	0.0709	22	KMT2D	0.0853
17	AKT2 (CNV)	0.162	11	NUMA1 (CNV)	0.188	17	*snoU13.ENSG... (CNV)	0.0688	73	*NCKAP5 (CNV)	0.0851
18	FGFR3 (CNV)	0.146	16	CBLB (CNV)	0.186	18	*SSPO	0.0665	20	*VPS13D	0.081
19	BIRC3 (CNV)	0.141	14	EGFR (CNV)	0.181	19	*DCC	0.0661	212	*RYR1	0.081
20	SETBP1 (CNV)	0.136	17	AKT2 (CNV)	0.175	20	*VPS13D	0.0635	21	*PGAP3 (CNV)	0.0808

Table S9: Top MSigDB C2.CP pathway models for Lower Grade Glioma

Consensus cancer genes			All TCGA genes		
#	Pathway	R^2_{pred}	#	Pathway	R^2_{pred}
1	PID_IL3_PATHWAY	0.471	5	KEGG_MISMATCH_REPAIR	0.463
2	PID_AR_NONGENOMIC_PATHWAY	0.46	2	PID_AR_NONGENOMIC_PATHWAY	0.461
3	KEGG_LEUKOCYTE_TRANSENDOTHELIAL_MIGRATION	0.454	1	PID_IL3_PATHWAY	0.457
4	REACTOME_ACTIVATION_OF_ATR_IN_RESPONSE_TO_REPLICAT...	0.441	3	KEGG_LEUKOCYTE_TRANSENDOTHELIAL_MIGRATION	0.449
5	KEGG_MISMATCH_REPAIR	0.433	4	REACTOME_ACTIVATION_OF_ATR_IN_RESPONSE_TO_REPLICAT...	0.447
6	KEGG_B_CELL_RECEPTOR_SIGNALING_PATHWAY	0.432	19	REACTOME_REPAIR_SYNTHESIS_FOR_GAP_FILLING_BY_DNA_P...	0.444
7	BIOCARTA_CDMAC_PATHWAY	0.432	6	KEGG_B_CELL_RECEPTOR_SIGNALING_PATHWAY	0.431
8	KEGG_PANTOTHENATE_AND_COA_BIOSYNTHESIS	0.431	23	KEGG_HOMOLOGOUS_RECOMBINATION	0.428
9	PID_ERA_GENOMIC_PATHWAY	0.428	8	KEGG_PANTOTHENATE_AND_COA_BIOSYNTHESIS	0.425
10	KEGG_GLYCOSAMINOGLYCAN_BIOSYNTHESIS_KERATAN_SULFAT...	0.425	9	PID_ERA_GENOMIC_PATHWAY	0.423
11	KEGG_FC_GAMMA_R_MEDIATED_PHAGOCYTOSIS	0.422	12	REACTOME_G2_M_CHECKPOINTS	0.423
12	REACTOME_G2_M_CHECKPOINTS	0.418	10	KEGG_GLYCOSAMINOGLYCAN_BIOSYNTHESIS_KERATAN_SULFAT...	0.42
13	PID_GMCSF_PATHWAY	0.414	40	REACTOME_PROCESSIVE_SYNTHESIS_ON_THE_LAGGING_STRAN...	0.419
14	BIOCARTA_RHO_PATHWAY	0.404	17	BIOCARTA_CASPASE_PATHWAY	0.417
15	BIOCARTA_UCALPAIN_PATHWAY	0.401	11	KEGG_FC_GAMMA_R_MEDIATED_PHAGOCYTOSIS	0.417
16	BIOCARTA_BCR_PATHWAY	0.401	39	REACTOME_LAGGING_STRAND_SYNTHESIS	0.417
17	BIOCARTA_CASPASE_PATHWAY	0.4	37	REACTOME_REMOVAL_OF_THE_FLAP_INTERMEDIATE_FROM_THE...	0.417
18	REACTOME_TRIGLYCERIDE_BIOSYNTHESIS	0.4	36	KEGG_DNA_REPLICATION	0.415
19	REACTOME_REPAIR_SYNTHESIS_FOR_GAP_FILLING_BY_DNA_P...	0.4	13	PID_GMCSF_PATHWAY	0.412
20	PID_ANTHRAX_PATHWAY	0.398	24	REACTOME_DNA_STRAND_ELONGATION	0.412

Table S10: Top MSigDB C6 pathway models for Lower Grade Glioma

Consensus cancer genes			All TCGA genes		
#	Pathway	R^2_{pred}	#	Pathway	R^2_{pred}
1	CORDENONSI_YAP_CONSERVED_SIGNATURE	0.365	2	RB_P107_DN.V1_UP	0.38
2	RB_P107_DN.V1_UP	0.361	1	CORDENONSI_YAP_CONSERVED_SIGNATURE	0.367
3	P53_DN.V2_DN	0.322	4	KRAS.50_UP.V1_UP	0.336
4	KRAS.50_UP.V1_UP	0.322	3	P53_DN.V2_DN	0.325
5	SNF5_DN.V1_UP	0.315	5	SNF5_DN.V1_UP	0.324
6	PRC2_EZH2_UP.V1_UP	0.314	7	SRC_UP.V1_DN	0.314
7	SRC_UP.V1_DN	0.306	6	PRC2_EZH2_UP.V1_UP	0.309
8	CAHOY_ASTROGLIAL	0.295	8	CAHOY_ASTROGLIAL	0.297
9	GCNP_SHH_UP_EARLY.V1_DN	0.291	10	KRAS.KIDNEY_UP.V1_UP	0.295
10	KRAS.KIDNEY_UP.V1_UP	0.29	9	GCNP_SHH_UP_EARLY.V1_DN	0.285
11	CRX_DN.V1_UP	0.282	12	KRAS.300_UP.V1_UP	0.281
12	KRAS.300_UP.V1_UP	0.28	14	NRL_DN.V1_UP	0.268
13	RPS14_DN.V1_DN	0.28	11	CRX_DN.V1_UP	0.265
14	NRL_DN.V1_UP	0.271	22	CRX_NRL_DN.V1_DN	0.263
15	CSR_LATE_UP.V1_UP	0.269	21	LEF1_UP.V1_UP	0.261
16	P53_DN.V1_UP	0.264	13	RPS14_DN.V1_DN	0.26
17	GLI1_UP.V1_DN	0.262	20	STK33_NOMO_DN	0.258
18	CSR_LATE_UP.V1_DN	0.259	17	GLI1_UP.V1_DN	0.258
19	CRX_NRL_DN.V1_UP	0.251	16	P53_DN.V1_UP	0.257
20	STK33_NOMO_DN	0.246	23	CRX_DN.V1_DN	0.255

Table S11: Top predictors for Lower Grade Glioma

Consensus cancer genes						All TCGA genes					
C2.CP			C6			C2.CP			C6		
#	Predictor	W	#	Predictor	W	#	Predictor	W	#	Predictor	W
1	**TP53**	0.671	1	**TP53**	0.627	1	**TP53**	0.639	1	**TP53**	0.674
2	ATRX	0.336	4	CDKN2A (CNV)	0.245	2	*RP11.156P1.3	0.288	2	*RP11.156P1.3	0.49
3	**EGFR (CNV)**	0.335	6	IDH1	0.207	3	ATRX	0.26	5	IDH1	0.186
4	CDKN2A (CNV)	0.249	3	**EGFR (CNV)**	0.18	4	**NF1**	0.187	14	*UBBP4	0.157
5	**NF1**	0.222	2	ATRX	0.168	5	IDH1	0.179	18	*NIPBL	0.139
6	IDH1	0.2	26	TSHR (CNV)	0.151	6	**CIC**	0.162	15	PIK3CA	0.128
7	MDM4 (CNV)	0.18	17	FGFR2 (CNV)	0.141	7	*FRG1B	0.161	11	*TTN	0.12
8	NOTCH1	0.18	5	**NF1**	0.138	8	*C11orf58 (CNV)	0.157	8	*C11orf58 (CNV)	0.119
9	**CIC**	0.175	12	PIK3CA	0.133	9	*PRSS3P2	0.142	4	**NF1**	0.118
10	CDK4 (CNV)	0.154	16	FBXW7 (CNV)	0.132	10	NOTCH1	0.139	12	*DNM1P47	0.113
11	**FUBP1**	0.143	32	RAD51B (CNV)	0.124	11	*TTN	0.135	7	*FRG1B	0.104
12	PIK3CA	0.142	14	GATA3 (CNV)	0.1	12	*DNM1P47	0.126	16	*BMS1P20	0.098
13	BCL9 (CNV)	0.141	11	**FUBP1**	0.0971	13	*AC024560.3	0.119	3	ATRX	0.0976
14	GATA3 (CNV)	0.137	57	ETNK1 (CNV)	0.0956	14	*UBBP4	0.117	13	*AC024560.3	0.0938
15	**PTEN**	0.133	10	CDK4 (CNV)	0.0954	15	PIK3CA	0.116	28	*EPHA5 (CNV)	0.0894
16	FBXW7 (CNV)	0.126	13	BCL9 (CNV)	0.0894	16	*BMS1P20	0.106	20	*FAM47C	0.0885
17	FGFR2 (CNV)	0.123	27	TET2 (CNV)	0.0877	17	**FUBP1**	0.105	35	*LANCL2 (CNV)	0.0868
18	ACKR3 (CNV)	0.114	29	CCND2 (CNV)	0.0866	18	*NIPBL	0.0948	21	*AQP7	0.0865
19	PDGFRA (CNV)	0.113	7	MDM4 (CNV)	0.0826	19	*C12orf40 (CNV)	0.0792	29	*MUC17	0.0832
20	FH (CNV)	0.109	8	NOTCH1	0.082	20	*FAM47C	0.0715	17	**FUBP1**	0.0751

Table S12: Top MSigDB C2.CP pathway models for Lung Adenocarcinoma

Consensus cancer genes			All TCGA genes		
#	Pathway	R^2_{pred}	#	Pathway	R^2_{pred}
1	BIOCARTA_RANMS_PATHWAY	0.487	1	BIOCARTA_RANMS_PATHWAY	0.465
2	REACTOME_ACTIVATION_OF_ATR_IN_RESPONSE_TO_REPLICAT...	0.486	3	REACTOME_G2_M_CHECKPOINTS	0.459
3	REACTOME_G2_M_CHECKPOINTS	0.483	2	REACTOME_ACTIVATION_OF_ATR_IN_RESPONSE_TO_REPLICAT...	0.456
4	REACTOME_CELL_CYCLE	0.472	6	REACTOME_CDC6_ASSOCIATION_WITH_THE_ORC_ORIGIN_COMP...	0.45
5	REACTOME_MITOTIC_M_M_G1_PHASES	0.468	14	REACTOME_CHROMOSOME_MAINTENANCE	0.434
6	REACTOME_CDC6_ASSOCIATION_WITH_THE_ORC_ORIGIN_COMP...	0.468	9	REACTOME_G0_AND_EARLY_G1	0.433
7	REACTOME_DNA_REPLICATION	0.464	4	REACTOME_CELL_CYCLE	0.431
8	REACTOME_CELL_CYCLE_MITOTIC	0.464	8	REACTOME_CELL_CYCLE_MITOTIC	0.429
9	REACTOME_G0_AND_EARLY_G1	0.454	5	REACTOME_MITOTIC_M_M_G1_PHASES	0.428
10	PID_ATR_PATHWAY	0.45	7	REACTOME_DNA_REPLICATION	0.427
11	KEGG_HOMOLOGOUS_RECOMBINATION	0.449	17	REACTOME_UNWINDING_OF_DNA	0.426
12	REACTOME_MITOTIC_PROMETAPHASE	0.448	15	REACTOME_ACTIVATION_OF_THE_PRE_REPLICATIVE_COMPLEX	0.42
13	REACTOME_E2F_ENABLED_INHIBITION_OF_PRE_REPLICATION...	0.447	10	PID_ATR_PATHWAY	0.419
14	REACTOME_CHROMOSOME_MAINTENANCE	0.446	11	KEGG_HOMOLOGOUS_RECOMBINATION	0.413
15	REACTOME_ACTIVATION_OF_THE_PRE_REPLICATIVE_COMPLEX	0.442	82	REACTOME_VITAMIN_B5_PANTOTHENATE_METABOLISM	0.411
16	KEGG_CELL_CYCLE	0.442	27	REACTOME_KINESINS	0.41
17	REACTOME_UNWINDING_OF_DNA	0.441	16	KEGG_CELL_CYCLE	0.407
18	PID_AURORA_B_PATHWAY	0.435	13	REACTOME_E2F_ENABLED_INHIBITION_OF_PRE_REPLICATION...	0.405
19	PID_FANCONI_PATHWAY	0.432	19	PID_FANCONI_PATHWAY	0.403
20	REACTOME_DNA_STRAND_ELONGATION	0.429	12	REACTOME_MITOTIC_PROMETAPHASE	0.401

Table S13: Top MSigDB C6 pathway models for Lung Adenocarcinoma

Consensus cancer genes			All TCGA genes		
#	Pathway	R^2_{pred}	#	Pathway	R^2_{pred}
1	PRC2_EZH2_UP.V1_UP	0.456	3	RPS14_DN.V1_DN	0.431
2	E2F1_UP.V1_UP	0.444	1	PRC2_EZH2_UP.V1_UP	0.413
3	RPS14_DN.V1_DN	0.441	7	KRAS.300_UP.V1_UP	0.411
4	HOXA9_DN.V1_DN	0.406	10	KRAS.600_UP.V1_UP	0.397
5	CSR_LATE_UP.V1_UP	0.396	2	E2F1_UP.V1_UP	0.382
6	PTEN_DN.V2_UP	0.385	8	CYCLIN_D1_KE_.V1_DN	0.376
7	KRAS.300_UP.V1_UP	0.381	6	PTEN_DN.V2_UP	0.357
8	CYCLIN_D1_KE_.V1_DN	0.376	111	NFE2L2.V2	0.355
9	RB_P107_DN.V1_UP	0.37	12	RPS14_DN.V1_UP	0.354
10	KRAS.600_UP.V1_UP	0.366	11	LEF1_UP.V1_DN	0.353
11	LEF1_UP.V1_DN	0.355	5	CSR_LATE_UP.V1_UP	0.353
12	RPS14_DN.V1_UP	0.351	14	PRC2_SUZ12_UP.V1_DN	0.348
13	P53_DN.V1_DN	0.349	4	HOXA9_DN.V1_DN	0.346
14	PRC2_SUZ12_UP.V1_DN	0.344	13	P53_DN.V1_DN	0.344
15	PDGF_UP.V1_DN	0.337	21	IL2_UP.V1_DN	0.339
16	GCNP_SHH_UP_EARLY.V1_UP	0.327	9	RB_P107_DN.V1_UP	0.321
17	GCNP_SHH_UP_LATE.V1_UP	0.325	29	KRAS.PROSTATE_UP.V1_UP	0.318
18	KRAS.LUNG_UP.V1_UP	0.322	19	IL15_UP.V1_DN	0.304
19	IL15_UP.V1_DN	0.321	15	PDGF_UP.V1_DN	0.304
20	E2F1_UP.V1_DN	0.319	16	GCNP_SHH_UP_EARLY.V1_UP	0.303

Table S14: Top predictors for Lung Adenocarcinoma

Consensus cancer genes						All TCGA genes					
C2.CP			C6			C2.CP			C6		
#	Predictor	W	#	Predictor	W	#	Predictor	W	#	Predictor	W
1	**TP53**	0.784	1	**TP53**	0.672	1	**KEAP1**	0.766	1	**KEAP1**	0.942
2	**SMARCA4**	0.559	3	MET (CNV)	0.59	2	**TP53**	0.484	2	**TP53**	0.423
3	MET (CNV)	0.445	2	**SMARCA4**	0.541	3	**SMARCA4**	0.339	4	**KRAS**	0.397
4	**KRAS**	0.374	4	**KRAS**	0.507	4	**KRAS**	0.249	3	**SMARCA4**	0.298
5	SETD2	0.268	8	**EGFR (CNV)**	0.319	5	**STK11**	0.171	8	*BAGE2	0.209
6	**RBM10**	0.263	25	**EGFR**	0.279	6	*DST	0.166	7	*FRG1B	0.148
7	**STK11**	0.239	9	FOXA1 (CNV)	0.262	7	*FRG1B	0.145	13	MET (CNV)	0.146
8	**EGFR (CNV)**	0.22	10	MYC (CNV)	0.241	8	*BAGE2	0.131	43	*PKHD1	0.137
9	FOXA1 (CNV)	0.213	44	EP300 (CNV)	0.229	9	*SPTA1	0.127	36	**EGFR**	0.13
10	MYC (CNV)	0.209	11	**SMARCA4 (CNV)**	0.227	10	*ANK2	0.115	6	*DST	0.127
11	**SMARCA4 (CNV)**	0.194	35	YWHAE (CNV)	0.224	11	*ZAN	0.111	22	*TENM1	0.125
12	CDKN2A (CNV)	0.191	17	**TPM3 (CNV)**	0.223	12	*RP1L1	0.109	9	*SPTA1	0.123
13	PIK3CA	0.19	114	VTI1A (CNV)	0.212	13	MET (CNV)	0.102	14	*AFF2	0.119
14	ATM	0.187	6	**RBM10**	0.212	14	*AFF2	0.0999	53	*NCKAP5	0.0982
15	**KRAS (CNV)**	0.18	32	NCOR2	0.208	15	*ARFGEF1	0.0954	92	*KCNH8	0.0959
16	CCNE1 (CNV)	0.177	24	COL1A1	0.208	16	*PGK2	0.0942	20	*SNHG14	0.0928
17	**TPM3 (CNV)**	0.168	51	ITK	0.208	17	SETD2	0.0893	5	**STK11**	0.0911
18	SBDS (CNV)	0.163	5	SETD2	0.206	18	*PGBD1	0.0856	37	*ANKRD30A	0.0895
19	CDK4 (CNV)	0.16	22	KMT2D	0.194	19	*UNC79	0.084	31	*SCN5A	0.0889
20	GNAS	0.16	12	CDKN2A (CNV)	0.191	20	*SNHG14	0.0837	260	*NBPF1	0.0888

Table S15: Top MSigDB C2.CP pathway models for Thyroid Cancer

Consensus cancer genes			All TCGA genes		
#	Pathway	R^2_{pred}	#	Pathway	R^2_{pred}
1	PID_HNF3B_PATHWAY	0.406	2	KEGG_GLYCEROLIPID_METABOLISM	0.41
2	KEGG_GLYCEROLIPID_METABOLISM	0.406	1	PID_HNF3B_PATHWAY	0.396
3	REACTOME_CELL_CELL_COMMUNICATION	0.405	3	REACTOME_CELL_CELL_COMMUNICATION	0.392
4	KEGG_PRIMARY_BILE_ACID_BIOSYNTHESIS	0.382	5	REACTOME_REGULATION_OF_COMPLEMENT_CASCADE	0.388
5	REACTOME_REGULATION_OF_COMPLEMENT_CASCADE	0.378	4	KEGG_PRIMARY_BILE_ACID_BIOSYNTHESIS	0.381
6	PID_ECADHERIN_STABILIZATION_PATHWAY	0.376	6	PID_ECADHERIN_STABILIZATION_PATHWAY	0.373
7	ST_TYPE_I_INTERFERON_PATHWAY	0.368	12	REACTOME_CELL_JUNCTION_ORGANIZATION	0.371
8	KEGG_GLYCINE_SERINE_AND_THREONINE_METABOLISM	0.368	10	REACTOME_TIGHT_JUNCTION_INTERACTIONS	0.369
9	PID_VEGF_VEGFR_PATHWAY	0.366	9	PID_VEGF_VEGFR_PATHWAY	0.36
10	REACTOME_TIGHT_JUNCTION_INTERACTIONS	0.364	7	ST_TYPE_I_INTERFERON_PATHWAY	0.358
11	KEGG_MATURITY_ONSET_DIABETES_OF_THE_YOUNG	0.363	11	KEGG_MATURITY_ONSET_DIABETES_OF_THE_YOUNG	0.354
12	REACTOME_CELL_JUNCTION_ORGANIZATION	0.359	18	REACTOME_INTRINSIC_PATHWAY	0.349
13	ST_INTERFERON_GAMMA_PATHWAY	0.358	14	KEGG_BUTANOATE_METABOLISM	0.344
14	KEGG_BUTANOATE_METABOLISM	0.355	8	KEGG_GLYCINE_SERINE_AND_THREONINE_METABOLISM	0.34
15	ST_STAT3_PATHWAY	0.354	17	BIOCARTA_AHSP_PATHWAY	0.335
16	REACTOME_TRIGLYCERIDE_BIOSYNTHESIS	0.349	16	REACTOME_TRIGLYCERIDE_BIOSYNTHESIS	0.335
17	BIOCARTA_AHSP_PATHWAY	0.334	29	REACTOME_INTEGRATION_OF_PROVIRUS	0.334
18	REACTOME_INTRINSIC_PATHWAY	0.334	24	REACTOME_EARLY_PHASE_OF_HIV_LIFE_CYCLE	0.327
19	PID_TNF_PATHWAY	0.331	25	REACTOME_CELL_CELL_JUNCTION_ORGANIZATION	0.325
20	KEGG_PPAR_SIGNALING_PATHWAY	0.324	15	ST_STAT3_PATHWAY	0.325

Table S16: Top MSigDB C6 pathway models for Thyroid Cancer

Consensus cancer genes			All TCGA genes		
#	Pathway	R^2_{pred}	#	Pathway	R^2_{pred}
1	SINGH_KRAS_DEPENDENCY_SIGNATURE_	0.42	1	SINGH_KRAS_DEPENDENCY_SIGNATURE_	0.432
2	P53_DN.V1_UP	0.399	2	P53_DN.V1_UP	0.404
3	ESC_V6.5_UP_EARLY.V1_DN	0.36	4	CAMP_UP.V1_DN	0.345
4	CAMP_UP.V1_DN	0.344	3	ESC_V6.5_UP_EARLY.V1_DN	0.339
5	STK33_SKM_DN	0.312	7	HINATA_NFKB_MATRIX	0.31
6	E2F1_UP.V1_DN	0.302	6	E2F1_UP.V1_DN	0.302
7	HINATA_NFKB_MATRIX	0.3	5	STK33_SKM_DN	0.293
8	MYC_UP.V1_DN	0.267	9	EGFR_UP.V1_UP	0.266
9	EGFR_UP.V1_UP	0.257	10	AKT_UP.V1_UP	0.264
10	AKT_UP.V1_UP	0.254	12	AKT_UP_MTOR_DN.V1_UP	0.25
11	CRX_DN.V1_DN	0.249	8	MYC_UP.V1_DN	0.245
12	AKT_UP_MTOR_DN.V1_UP	0.242	11	CRX_DN.V1_DN	0.242
13	SRC_UP.V1_DN	0.238	14	PTEN_DN.V2_DN	0.236
14	PTEN_DN.V2_DN	0.23	16	ESC_J1_UP_EARLY.V1_DN	0.234
15	RAF_UP.V1_UP	0.229	15	RAF_UP.V1_UP	0.231
16	ESC_J1_UP_EARLY.V1_DN	0.227	18	MEK_UP.V1_UP	0.228
17	CAHOY_ASTROGLIAL	0.224	19	BMI1_DN.V1_UP	0.227
18	MEK_UP.V1_UP	0.22	21	ATF2_UP.V1_DN	0.214
19	BMI1_DN.V1_UP	0.22	20	IL2_UP.V1_UP	0.209
20	IL2_UP.V1_UP	0.212	13	SRC_UP.V1_DN	0.208

Table S17: Top predictors for Thyroid Cancer

Consensus cancer genes						All TCGA genes					
C2.CP			C6			C2.CP			C6		
#	Predictor	W	#	Predictor	W	#	Predictor	W	#	Predictor	W
1	**BRAF**	1.5	1	**BRAF**	1.59	1	**BRAF**	1.68	1	**BRAF**	1.72
2	**NRAS**	0.521	2	**NRAS**	0.576	2	**NRAS**	0.474	2	**NRAS**	0.483
3	HRAS	0.335	3	HRAS	0.269	3	HRAS	0.304	3	HRAS	0.235
4	RB1 (CNV)	0.0818	5	**KRAS**	0.0622	4	*Unknown	0.136	4	*Unknown	0.186
5	**KRAS**	0.0603	4	RB1 (CNV)	0.0588	5	*HSD17B7P2	0.114	9	*ADAM6	0.17
6	SF3B1	0.0594	23	PTPN11 (CNV)	0.0582	6	*C21orf99	0.0991	7	*DNAH9	0.105
7	ATM	0.0482	7	ATM	0.0484	7	*DNAH9	0.0845	12	*FLJ36000	0.0962
8	CLTCL1 (CNV)	0.0434	19	SMARCB1 (CNV)	0.0481	8	*MST1P2	0.0767	8	*MST1P2	0.092
9	LZTR1 (CNV)	0.0434	52	EWSR1 (CNV)	0.0397	9	*ADAM6	0.059	18	*EIF1AX	0.0906
10	GNAS	0.0429	13	AKT1	0.0388	10	*LOC442421	0.0582	5	*HSD17B7P2	0.0884
11	PDE4DIP	0.0427	28	JAK2 (CNV)	0.0375	11	*FRG1B	0.0552	21	*LOC284232	0.0844
12	MALAT1	0.0416	21	ARID1B	0.0373	12	*FLJ36000	0.0524	19	*LOC374491	0.0793
13	AKT1	0.039	31	NRG1 (CNV)	0.0367	13	*GOLGA6L10	0.0522	55	*FAM182B	0.0673
14	WRN	0.0388	36	ERBB4	0.0346	14	*TUBBP5	0.0518	79	*ITPR2	0.0562
15	KDR	0.0372	34	MET (CNV)	0.0338	15	*ZBTB22	0.0517	13	*GOLGA6L10	0.0518
16	CACNA1D	0.0359	6	SF3B1	0.0321	16	*WASH3P	0.0472	75	*BOD1L1	0.051
17	IL7R	0.0355	48	**DICER1**	0.032	17	*CECR2 (CNV)	0.0444	34	*RPL23AP53	0.0482
18	KAT6B	0.0352	33	**NTRK1 (CNV)**	0.0275	18	*EIF1AX	0.044	54	*LOC644669	0.0459
19	SMARCB1 (CNV)	0.0343	9	LZTR1 (CNV)	0.0231	19	*LOC374491	0.0431	35	*LOC220729	0.0437
20	PIK3CA	0.0341	8	CLTCL1 (CNV)	0.0221	20	RB1 (CNV)	0.0416	11	*FRG1B	0.0427

Table S18: MSigDB C2.CP pathways differentially active in luminal vs. basal breast cancer

Upregulated in luminal		Upregulated in basal	
Pathway	Statistic	Pathway	Statistic
REACTOME_SIGNALING_BY_PDGF	14	REACTOME_REGULATION_OF_MITOTIC_CELL_CYCLE	-17
REACTOME_NRAGE_SIGNALS_DEATH_THROUGH_JNK	13	REACTOME_NEF_MEDIATED_DOWNREGULATION_OF_MHC_CLASS_I...	-17
REACTOME_GABA_SYNTHESIS_RELEASE_REUPTAKE_AND_DEGRADATION	13	REACTOME_P75NTR_RECRUITS_SIGNALLING_COMPLEXES	-16
BIOCARTA_LEPTIN_PATHWAY	13	REACTOME_E2F_ENABLED_INHIBITION_OF_PRE_REPLICATION_COMPLEX...	-16
BIOCARTA_FIBRINOLYSIS_PATHWAY	13	KEGG_NATURAL_KILLER_CELL_MEDIATED_CYTOTOXICITY	-15
KEGG_GLYCOSPHINGOLIPID_BIOSYNTHESIS_GANGLIO_SERIES	13	REACTOME_RNA_POL_I_TRANSCRIPTION	-14
SA_G2_AND_M_PHASES	13	BIOCARTA_G2_PATHWAY	-14
KEGG_RIG_I_LIKE_RECEPTOR_SIGNALING_PATHWAY	13	KEGG_PATHOGENIC_ESCHERICHIA_COLI_INFECTION	-13
KEGG_NOD_LIKE_RECEPTOR_SIGNALING_PATHWAY	12	REACTOME_IRAK2_MEDIATED_ACTIVATION_OF_TAK1_COMPLEX_UPON_TLR7...	-13
PID_AVB3_INTEGRIN_PATHWAY	12	REACTOME_UNFOLDED_PROTEIN_RESPONSE	-13

Table S19: MSigDB C6 pathways differentially active in luminal vs. basal breast cancer

Upregulated in luminal		Upregulated in basal	
Pathway	Statistic	Pathway	Statistic
PIGF_UP.V1_UP	13	VEGF_A_UP.V1_DN	-14
KRAS.DF.V1_UP	11	SRC_UP.V1_DN	-12
KRAS.LUNG_UP.V1_UP	10	ATF2_S_UP.V1_UP	-11
JNK_DN.V1_UP	10	KRAS.DF.V1_DN	-11
BCAT_BILD_ET_AL_DN	9.2	MTOR_UP.N4.V1_UP	-9.4
MTOR_UP.V1_DN	8.9	RB_P107_DN.V1_UP	-9.4
CAHOY_NEURONAL	7.9	IL21_UP.V1_UP	-9
LTE2_UP.V1_UP	7.8	IL15_UP.V1_UP	-8.9
PDGF_UP.V1_DN	7.6	MEL18_DN.V1_UP	-8.8
YAP1_DN	7.5	E2F3_UP.V1_DN	-8.7

Table S20: Genes whose somatic mutation drives dysregulation of MSigDB C2.CP pathways. Relative importance in luminal vs. basal breast cancer.

Important in luminal		Important in basal	
Gene	Statistic	Gene	Statistic
PIK3CA	134	TP53	-603
CDH1	92	TTN	-26
GATA3	47	DNAH17	-3.4
MAP3K1	5.4	APOB	-2.6
MLL3	1.6	FLG	-2.6
CROCCP2	1.6	RB1	-0.99
FRG1B	0.53	DST	-0.61
MAP2K4	0.42	MUC16	-0.36
ZFHX4	0.18	MDN1	-0.34
CBFB	0.18	SYNE1	-0.29

Table S21: Genes whose somatic mutation drives dysregulation of MSigDB C6 pathways. Relative importance in luminal vs. basal breast cancer.

Important in luminal		Important in basal	
Gene	Statistic	Gene	Statistic
PIK3CA	24	TP53	-99
CDH1	20	TTN	-3.1
GATA3	7.8	APOB	-0.78
MAP3K1	0.38	DNAH17	-0.17
MLL3	0.35	FLG	-0.087
FRG1B	0.099	PEG3	-0.084
CROCCP2	0.093	RB1	-0.072
NBPF1	0.073	CSPP1	-0.064
CBFB	0.059	SPTA1	-0.062
KIAA0913	0.032	MUC16	-0.059

Table S22: Genes whose copy number variation drives dysregulation of MSigDB C2.CP pathways. Relative importance in luminal vs. basal breast cancer.

Important in luminal		Important in basal	
Gene	Statistic	Gene	Statistic
SHANK2	34	ERC1	-35
ZNF703	28	PVT1	-30
RNA5SP406	15	ETV6	-29
IKZF3	13	URI1	-22
HERC2P5	12	RN7SKP226	-21
RNA5SP409	12	FGF12	-18
RNA5SP407	12	CCDC171	-17
ZNF217	12	KLF6	-15
NOL11	11	CSMD3	-14
NEUROD2	11	ADIPOR2	-14

Table S23: Genes whose copy number variation drives dysregulation of MSigDB C6 pathways. Relative importance in luminal vs. basal breast cancer.

Important in luminal		Important in basal	
Gene	Statistic	Gene	Statistic
SHANK2	5.6	RN7SKP226	-4.7
ZNF217	3.1	PVT1	-4.6
NEUROD2	2.7	FAM208B	-3.5
IKZF3	2.5	ETV6	-3.1
RNA5SP406	2.5	FGF12	-3.1
ZNF703	2.4	ERC1	-2.9
HERC2P5	2.4	ADIPOR2	-2.6
RNA5SP409	2.2	SV2A	-2.5
NOL11	2.1	NOTCH2	-2.5
RNA5SP407	2.1	URI1	-2.2

Table S24: Top predictors for Pancreatic Cancer using GSVA

Consensus cancer genes						All TCGA genes					
C2.CP			C6			C2.CP			C6		
#	Predictor	W	#	Predictor	W	#	Predictor	W	#	Predictor	W
1	ERCC3	0.195	1	ERCC3	0.281	1	*MYH10	0.109	3	**KRAS**	0.184
2	CAMTA1 (CNV)	0.147	5	BLM	0.219	2	TP53	0.104	8	*NOX4	0.177
3	**CDKN2A (CNV)**	0.127	9	PTPN13	0.213	3	**KRAS**	0.0921	11	*FOXN3	0.113
4	ZFHX3	0.118	4	ZFHX3	0.178	4	*TCF20	0.0606	81	*FOXE1	0.106
5	BLM	0.11	3	**CDKN2A (CNV)**	0.175	5	*TUBB8P7	0.0606	25	*ZNRD1.AS1	0.0967
6	TP53	0.107	8	**CDKN2A**	0.163	6	*PRG4	0.0596	4	*TCF20	0.09
7	CHEK2	0.103	2	CAMTA1 (CNV)	0.136	7	*BTN2A3P	0.0579	23	*TRAPPC9 (CNV)	0.0874
8	**CDKN2A**	0.0985	10	**KRAS**	0.133	8	*NOX4	0.057	7	*BTN2A3P	0.084
9	PTPN13	0.086	33	FOXP1	0.133	9	*ANK3	0.0511	1	*MYH10	0.0836
10	**KRAS**	0.081	29	CBL	0.131	10	**CDKN2A**	0.0489	12	*TSSC2	0.0835
11	NCOR1 (CNV)	0.0794	7	CHEK2	0.13	11	*FOXN3	0.0488	10	**CDKN2A**	0.0818
12	RANBP2	0.0775	21	KMT2D	0.117	12	*TSSC2	0.0479	6	*PRG4	0.0787
13	TRIM33	0.0775	26	ERC1 (CNV)	0.106	13	*CROCCP2	0.0464	56	*RP11.114H24.7	0.0783
14	NDRG1 (CNV)	0.0763	20	NOTCH2 (CNV)	0.103	14	*EDEM1	0.0464	41	*ZNF733P	0.0767
15	TFE3	0.0727	15	TFE3	0.0985	15	*CASC8 (CNV)	0.045	146	*AQP7	0.0766
16	MLLT3	0.071	42	**FAT4**	0.0965	16	*RYR1	0.0441	9	*ANK3	0.0729
17	KDM5A (CNV)	0.0677	6	TP53	0.0936	17	*SIPA1L1	0.0432	61	*TAOK2	0.0708
18	DEK	0.0658	45	HMGN2P46	0.0886	18	*FRG1B	0.0429	137	*FAM66D	0.0696
19	**SMAD4**	0.0654	24	**MAP2K4 (CNV)**	0.0882	19	*SNORA74...(CNV)	0.0417	55	CHEK2	0.0671
20	NOTCH2 (CNV)	0.0652	84	EZR (CNV)	0.0836	20	*ATAD2	0.041	62	*CDKN2B (CNV)	0.0665

Table S25: Top predictors for Pancreatic Cancer using ssGSEA

Consensus cancer genes						All TCGA genes					
C2.CP			C6			C2.CP			C6		
#	Predictor	W	#	Predictor	W	#	Predictor	W	#	Predictor	W
1	CAMTA1 (CNV)	0.397	2	**KRAS**	0.567	1	**KRAS**	0.313	1	**KRAS**	0.615
2	**KRAS**	0.305	1	CAMTA1 (CNV)	0.433	2	*ANK3	0.162	57	*OTUD4	0.117
3	ERCC3	0.213	4	ZFHX3	0.218	3	*EEF1B2	0.148	2134	*NOX4	0.0927
4	ZFHX3	0.204	5	PTPN13	0.205	4	*FRG1B	0.098	55	*RP11.782C8.2	0.0888
5	PTPN13	0.191	6	KMT2D	0.193	5	TP53	0.0895	2105	*C9orf53 (CNV)	0.0816
6	KMT2D	0.162	3	ERCC3	0.192	6	*CROCCP2	0.077	50	*HERC2P3	0.0816
7	MECOM (CNV)	0.162	11	FOXP1	0.189	7	*FRG1	0.072	9	*RYR1	0.077
8	RANBP2	0.161	25	CHEK2	0.176	8	*EDEM1	0.069	12	*SPRED3	0.0733
9	AKAP9	0.141	13	**CDKN2A**	0.173	9	*RYR1	0.0683	25	*LRCH1	0.0703
10	**CDKN2A (CNV)**	0.135	10	**CDKN2A (CNV)**	0.157	10	*ZFP90	0.0613	23	*CCT6P1	0.0662
11	FOXP1	0.129	16	TP53	0.151	11	RANBP2	0.0595	2138	*BTN2A3P	0.0636
12	ZNF384 (CNV)	0.123	9	AKAP9	0.151	12	*SPRED3	0.0556	2147	*RMDN3	0.054
13	**CDKN2A**	0.122	26	NACA	0.148	13	*VWF (CNV)	0.0522	33	*TPTE2	0.0531
14	**SMAD4**	0.113	38	GNAS	0.125	14	*RP11.368J21.3	0.0522	2184	*KCNB1	0.0516
15	MLLT3	0.112	45	**FAT4**	0.124	15	*AC008103.5	0.0508	27	*MAMLD1	0.0509
16	TP53	0.112	28	CHD4 (CNV)	0.114	16	*NOB1	0.0496	46	*GTF2IRD2P1	0.0492
17	TRIM33	0.0981	23	ERC1 (CNV)	0.112	17	*PRG4	0.0484	2111	*ANKRD20A8P	0.049
18	CBL	0.0956	18	CBL	0.11	18	*NTF3 (CNV)	0.0475	2163	*AQP7	0.049
19	MKL1	0.0939	12	ZNF384 (CNV)	0.103	19	AKAP9	0.0468	2161	*NBPF10	0.0473
20	SLC34A2	0.0841	72	LIFR (CNV)	0.101	20	**CDKN2A**	0.0466	74	*PRPF8	0.0465

References

Barbie, D. A., Tamayo, P., Boehm, J. S., Kim, S. Y., Moody, S. E., Dunn, I. F., Schinzel, A. C., Sandy, P., Meylan, E., Scholl, C., Fröhling, S., Chan, E. M., Sos, M. L., Michel, K., Mermel, C., Silver, S. J., Weir, B. A., Reiling, J. H., Sheng, Q., Gupta, P. B., Wadlow, R. C., Le, H., Hoersch, S., Wittner, B. S., Ramaswamy, S., Livingston, D. M., Sabatini, D. M., Meyerson, M., Thomas, R. K., Lander, E. S., Mesirov, J. P., Root, D. E., Gilliland, D. G., Jacks, T., and Hahn, W. C. (2009). Systematic rna interference reveals that oncogenic kras-driven cancers require tbk1. *Nature*, **462**(7269), 108–12.

Benjamini, Y. and Hochberg, Y. (1995). Controlling the false discovery rate: a practical and powerful approach to multiple testing. *Journal of the Royal Statistical Society. Series B (Statistical Methodology)*, pages 289–300.

Cancer Genome Atlas Research Network, Weinstein, J. N., Collisson, E. A., Mills, G. B., Shaw, K. R. M., Ozenberger, B. A., Ellrott, K., Shmulevich, I., Sander, C., and Stuart, J. M. (2013). The cancer genome atlas pan-cancer analysis project. *Nat Genet*, **45**(10), 1113–20.

Forbes, S. A., Beare, D., Gunasekaran, P., Leung, K., Bindal, N., Boutselakis, H., Ding, M., Bamford, S., Cole, C., Ward, S., Kok, C. Y., Jia, M., De, T., Teague, J. W., Stratton, M. R., McDermott, U., and Campbell, P. J. (2015). Cosmic: exploring the world's knowledge of somatic mutations in human cancer. *Nucleic Acids Res*, **43**(Database issue), D805–11.

Friedman, J. H., Hastie, T., and Tibshirani, R. (2010). Regularization paths for generalized linear models via coordinate descent. *Journal of Statistical Software*, **33**(1), 1–22.

Goldman, M., Craft, B., Swatloski, T., Cline, M., Morozova, O., Diekhans, M., Haussler, D., and Zhu, J. (2015). The ucsc cancer genomics browser: update 2015. *Nucleic Acids Res*, **43**(Database issue), D812–7.

Hänzelmann, S., Castelo, R., and Guinney, J. (2013). Gsva: gene set variation analysis for microarray and rna-seq data. *BMC Bioinformatics*, **14**, 7.

Hoadley, K. A., Yau, C., Wolf, D. M., Cherniack, A. D., Tamborero, D., Ng, S., Leiserson, M. D. M., Niu, B., McLellan, M. D., Uzunangelov, V., Zhang, J., Kandoth, C., Akbani, R., Shen, H., Omberg, L., Chu, A., Margolin, A. A., Van't Veer, L. J., Lopez-Bigas, N., Laird, P. W., Raphael, B. J., Ding, L., Robertson, A. G., Byers, L. A., Mills, G. B., Weinstein, J. N., Van Waes, C., Chen, Z., Collisson, E. A., Cancer Genome Atlas Research Network, Benz, C. C., Perou, C. M., and Stuart, J. M. (2014). Multiplatform analysis of 12 cancer types reveals molecular classification within and across tissues of origin. *Cell*, **158**(4), 929–944.

Lee, E., Chuang, H.-Y., Kim, J.-W., Ideker, T., and Lee, D. (2008). Inferring pathway activity toward precise disease classification. *PLoS Comput Biol*, **4**(11), e1000217.

Liberzon, A., Subramanian, A., Pinchback, R., Thorvaldsdóttir, H., Tamayo, P., and Mesirov, J. P. (2011). Molecular signatures database (msigdb) 3.0. *Bioinformatics*, **27**(12), 1739–40.

Mermel, C. H., Schumacher, S. E., Hill, B., Meyerson, M. L., Beroukhim, R., and Getz, G. (2011). Gistic2.0 facilitates sensitive and confident localization of the targets of focal somatic copy-number alteration in human cancers. *Genome Biol*, **12**(4), R41.

Tibshirani, R. (2011). Regression shrinkage and selection via the lasso: a retrospective. *Journal of the Royal Statistical Society. Series B (Statistical Methodology)*, **73**(Part 3), 273–282.

Tomfohr, J., Lu, J., and Kepler, T. B. (2005). Pathway level analysis of gene expression using singular value decomposition. *BMC Bioinformatics*, **6**, 225.
